# Supplementary material for: Vitrectomy combined with lens capsule flap transplantation in the treatment of high myopia macular hole retinal detachment: study protocol for a prospective randomised controlled trial
Source: BMJ Open. 2022 Jul 28;12(7):e064299. doi: 10.1136/bmjopen-2022-064299 (PMC9341187; doi:10.1136/bmjopen-2022-064299)
Supplement: Supplementary data [file bmjopen-2022-064299supp001.pdf]

Institutional Review Board of Shanghai General Hospital

Approval Letter of Shanghai General Hospital Institutional Review Board

<table>
<tr><td>Approval No: 2022SQ284.1</td><td>Review date: June 24, 2022</td><td colspan="2">Project No: 2022SQ284.1</td></tr>
<tr><td>Study Protocol</td><td colspan="3">A prospective randomized controlled trial of vitrectomy combined with lens capsule flap transplantation in the treatment of high myopia macular hole retinal detachment</td></tr>
<tr><td>Project source</td><td colspan="3">Initiated by researchers</td></tr>
<tr><td>Principal investigator</td><td colspan="3">Tianwei Qian</td></tr>
<tr><td>Research Center</td><td>Shanghai General Hospital</td><td>Department</td><td>Ophthalmology</td></tr>
<tr><td colspan="4">Review documents (including version number)
(1) Study protocol (version: 1.1)
(2) Resume of principal investigator (version: 1.0)
(3) Informed consent (version: 1.1)</td></tr>
</table>

1. Review method

Rapid review

2. Results

Agree

3. The study will be subject to ongoing review by the ethics committee?

■ **Yes** ☐**No**

The censorship frequency is once every 12 months from the date of approval. The ethics committee has the right to change the frequency of continuous review according to the actual situation.

Institutional Review Board

Date: June 24, 2022

Address: No.100 Haining Road, Shanghai **Official E-mail:** shiyilunli@sina.com
