# Supplementary material for: Vitrectomy combined with lens capsule flap transplantation in the treatment of high myopia macular hole retinal detachment: study protocol for a prospective randomised controlled trial
Source: BMJ Open. 2022 Jul 28;12(7):e064299. doi: 10.1136/bmjopen-2022-064299 (PMC9341187; doi:10.1136/bmjopen-2022-064299)
Supplement: Supplementary data [file bmjopen-2022-064299supp002.pdf]

Informed Consent Notification Page

Trial: A prospective randomized controlled trial of vitrectomy combined with lens capsule flap transplantation in the treatment of high myopia macular hole retinal detachment

Sponsor：Shanghai General Hospital

We invite you to participate in a study: A prospective randomized controlled trial of vitrectomy combined with lens capsule flap transplantation in the treatment of high myopia macular hole retinal detachment. Before you decide to participate in this trial, please read this informed consent carefully. If you have any questions you don't understand, you can ask the researcher in charge of the trial or members of the trial working group to explain any terms or materials you don't know.

1. Research background and purpose

1.1 Research background

Vitrectomy combined with internal limiting membrane (ILM) peeling, flap or tamponade is widely used in the treatment of macular diseases, such as macular hole (MH) and high myopia macular hole retinal detachment (HMMHRD). However, movement of the ILM to a suitable position to prevent displacement is a difficult operation. Improving visual function after surgery remains controversial. Compared to ILM, the thicker and more flexible lens capsule is easy to obtain and operate. Previous studies have confirmed the effectiveness of lens capsule flap in the treatment of MH.

1.2 Research purpose

This study aims to evaluate the efficacy and safety of vitrectomy combined with lens capsule flap transplantation in the treatment of HMMHRD.

2. Methods

2.1 Who can take part in the trial?

The inclusion criteria are as follows:

1） Patients ≥ 50 years old who were diagnosed as HMMHRD by indirect ophthalmoscopy, macular OCT, fundus photography, and B-ultrasound examination;
2） equivalent spherical dioptre ≤ –6.00 D or axis length ≥ 26 mm;
3） MH as main cause of RD;
4） turbid lens without obvious calcification in the lens capsule, requiring combination with cataract surgery;
5） the range of RD within the retinal vascular arch of the posterior pole;
6） consent to undergo surgical treatment and regular follow-up and sign relevant informed consent.

2.2 Who can't take part in the experiment?

1） patients with RD with other types of holes except MH, choroidal detachment, obvious proliferative vitreoretinopathy, intraocular haemorrhage, severe cataract, glaucoma, retinal vascular inflammation or obstruction, macular degeneration, macular neovascularization, macular haemorrhage, pathological myopia, and macular atrophy;
2） patients with previous history of intraocular surgery (including PPV and intravitreal drug injection)
3） patients whose general condition cannot tolerate the operation;
4） patients who cannot cooperate with the examination and follow-up for <6 months;
5） patients unwilling to undergo concurrent vitrectomy and cataract surgery;
6） patients with concomitant diseases or conditions of the target eye or whole body (including malignant hypertension; AIDS; malignant tumour; serious mental, cardiovascular, neurologic, respiratory, digestive, or other systemic disease; long-term use of hormones; and immunodeficiency after heart stenting or organ transplantation);
7） patients with diabetes with poor blood glucose control;
8） patients with poor communication skills and compliance;
9） patients without the ability to follow the postoperative position;
10） participation in other trials.

2.3 Trial design and sample size

The prospective, single-centre RCT will enrol 54 participants that will be randomised in a 1:1 ratio to the experimental group (vitrectomy combined with lens capsule flap transplantation) or control group (vitrectomy combined with ILM tamponade).

2.4 Interventions

2.4.1 Experimental group

Patients will first undergo phacoemulsification and intraocular lens implantation. During this operation, the lens capsule flap will be obtained and placed in sterile distilled water for at least 30 minutes. Thereafter, the 23-gauge vitrectomy through the pars plana will be performed, and the ILM will be stained with brilliant blue. After brilliant blue staining, the ILM in the macular area will be completely removed with intraocular forceps. Then the small piece of lens capsule flap is moved into the vitreous cavity with microforceps trough the 23-gauge trocar. The trimming of lens capsule flap will be performed in the vitreous cavity, and the lens capsule flap can be directly placed above the MH surface. The capsular flap should be trimmed by vitrectomy cutter as round as possible, and its diameter is about twice that of the macular hole. Afterwards, the lens capsule flap will need to be inserted into the macular hole to complete gas-liquid exchange. In this process, the operator will ensure that the capsule flap is always right under the macular hole. Finally, silicone oil (Oxane 5700 Bausch & Lomb, Kingston-upon-Thames, UK) will be injected into the vitreous cavity.

2.4.2 Control group

Patients will first undergo phacoemulsification and intraocular lens implantation. Thereafter, 23-gauge vitrectomy through the pars plana will be performed. After complete vitreous removal, ILM will be stained with brilliant blue. After brilliant blue staining, the ILM of the retina in the brilliant blue staining area will be directly clamped, and the ILM ring in the area will be removed about 1.5-disc diameters away from the macular hole. Care will be taken to prevent peeling off the ILM valve at the edge of the macular hole. Thereafter, the free ILM flap will be turned over and filled into the MH. During the operation, accurate filling of ILM into the MH will be confirmed severally. After complete gas-liquid exchange, silicone oil (Oxane 5700 Bausch & Lomb) will be injected into the vitreous cavity.

2.5 Research procedures

After fully informed and signed the informed consent, the subjects could enter the screening

period; In the screening period, the evaluation researchers evaluated whether they were included or not according to the inclusion/exclusion criteria. Only the qualified subjects can enter the study treatment period. The research doctor needs your cooperation in the implementation of relevant examination items to determine whether you meet the inclusion and exclusion criteria. These examinations are routine examinations for clinical diagnosis and treatment of this disease.

All participants who meet the inclusion criteria of this study and do not meet any of the exclusion criteria can be included in this study. After passing the screening, the participants who provided written informed consent will be randomly assigned to two parallel trial groups in a 1:1 ratio according to the central randomization system. The patients will undergo vitrectomy combined with lens capsule flap transplantation or ILM tamponade and regular post-surgery follow-up. Participants will be blinded to the specific surgical methods.

3. Research risks and benefits

3.1 Research risks

When your health condition is harmed by participating in this trial, the research doctor will take necessary medical measures. If you have any side effects or discomfort during the trial, it is essential that you report to the study doctor immediately. You may be given other drugs to control side effects. When participating in this study, you will need to provide some personal information, and we will take all necessary measures to ensure the confidentiality of the information.

3.2 Research benefits

Your participation may be helpful to further explore the efficacy and safety of vitrectomy combined with lens capsule flap transplantation in the treatment of HMMHRD.

4. Your rights

You have the right to decide whether to participate in the experiment. If you can't make a decision immediately, you have enough time to consider it. If necessary, you can discuss with relatives, friends and other people you trust before making a decision. If you decide not to participate in this trial, your relationship with the researcher and the sponsor will not be affected, you will not be discriminated against or retaliated, and your treatment and rights will not be affected. If you decide to participate in this trial, we hope you can complete the trial without special reasons, but you have the right to withdraw at any time during the trial. If you decide to quit, please let the researcher know in time.

During the experiment, you can know the information related to you at any time. If you have any

questions about this study, or if you feel unwell during the treatment, please contact the attending doctor. Contact doctor: Tianwei Qian 15201955168. And you have the right to consult about your rights or related risks.

5. Privacy and confidentiality

The personal information (such as name, gender, contact information, questionnaire, etc.) provided by you to the researcher may be known to the following persons or units in addition to the needs of normal research:

- Staff (inspectors, inspectors, etc.) of research funding institutions related to this experiment；
- State and local food and Drug Administration and other administrative agencies.

However, no one is allowed to disclose your personal information to others or other organizations without your permission. Except for researchers and administrative organizations, no other person or organization has the right to contact you about this experiment or provide you with information about this experiment directly.

The results of this experiment may be published in the form of academic papers, but your personal information will not appear in any publicly published documents.

6. Others

6.1 In case of the following situations, the researcher may withdraw you from the trial without your consent for your health:：

- If you continue to participate in this trial, your risk may outweigh your benefit;
- You did not follow the guidance of the researcher and did not participate in the trial according to the research plan;
- Early termination of trial.

6.2 This informed consent is in duplicate, one for the researcher and one for you.

7. Compensation for injury caused by trial

If your injury is directly caused by participating in this trial, you do not have to pay the medical expenses for treatment, which will be borne by the researcher.

Informed Consent Page

Consent statement:

1. I have read the instructions to the subjects carefully and understood the relevant background of this trial. The researchers have explained the characteristics of the study and possible adverse reactions to me in detail, and answered my questions.

2. I know that if I refuse to participate in this trial, my treatment and rights will not be affected. After understanding all the contents of the instructions to the subjects and having fully considered them, I voluntarily participate in this trial.

3. I am willing to follow the instructions of the researchers and participate in the trial according to the research protocol. During the experiment, I have the right to withdraw at any time, but before I withdraw, I need to inform the researchers in time.

4. During the trial, if there are any discomfort symptoms, I will tell the researchers in time.

Signature of Subject：

		/ /
Name (regular script)	Signature	Date

Signature of Researcher：

		/ /
Name (regular script)	Signature	Date

Signature of agent/Guardian (if any)：

The reason why the subject can't sign this page: ______________________

The relationship between the agent/guardian and the subject: ______________________

		/ /
Name (regular script)	Signature	Date
