# Supplementary material for: Vitrectomy combined with lens capsule flap transplantation in the treatment of high myopia macular hole retinal detachment: study protocol for a prospective randomised controlled trial
Source: BMJ Open. 2022 Jul 28;12(7):e064299. doi: 10.1136/bmjopen-2022-064299 (PMC9341187; doi:10.1136/bmjopen-2022-064299)
Supplement: Author's manuscript [file bmjopen-2022-064299.draft_revisions.pdf]

BMJ Open

BMJ Open is committed to open peer review. As part of this commitment we make the peer review history of every article we publish publicly available.

When an article is published we post the peer reviewers' comments and the authors' responses online. We also post the versions of the paper that were used during peer review. These are the versions that the peer review comments apply to.

The versions of the paper that follow are the versions that were submitted during the peer review process. They are not the versions of record or the final published versions. They should not be cited or distributed as the published version of this manuscript.

BMJ Open is an open access journal and the full, final, typeset and author-corrected version of record of the manuscript is available on our site with no access controls, subscription charges or pay-per-view fees (http://bmjopen.bmj.com).

If you have any questions on BMJ Open's open peer review process please email info.bmjopen@bmj.com

BMJ Open

Vitrectomy combined with lens capsule flap transplantation in the treatment of high myopia macular hole retinal detachment: study protocol for a prospective randomized controlled trial

Journal:	*BMJ Open*
Manuscript ID	bmjopen-2022-064299
Article Type:	Protocol
Date Submitted by the Author:	28-Apr-2022
Complete List of Authors:	Gong, Qiaoyun; Shanghai General Hospital, Department of Ophthalmology<br>Ye, Luyao; Shanghai General Hospital, Department of Ophthalmology<br>Wu, Xia; Shanghai General Hospital, Department of Ophthalmology<br>Xue, Lin; Shanghai General Hospital, Department of Ophthalmology<br>Zhou, Hao; Shanghai General Hospital, Department of Ophthalmology<br>Fan, Ying; Shanghai General Hospital, Department of Ophthalmology<br>Xu, Xun; Shanghai General Hospital, Ophthalmology<br>Wang, Weijun; Shanghai General Hospital, Department of Ophthalmology<br>Qian, Tianwei; Shanghai General Hospital, Department of Ophthalmology
Keywords:	Vetreoretinal < OPHTHALMOLOGY, Ophthalmology < SURGERY, Medical retina < OPHTHALMOLOGY

SCHOLARONE™
Manuscripts

Vitrectomy combined with lens capsule flap transplantation in the treatment of high myopia macular hole retinal detachment: study protocol for a prospective randomized controlled trial

Qiaoyun Gong[1-5], Luyao Ye[1-5], Xia Wu[1-5], Lin Xue[1-5], Hao Zhou[1-5], Ying Fan[1-5], Xun Xu[1-5], Weijun Wang[1-5], Tianwei Qian[1-5]

[1] Department of Ophthalmology, Shanghai General Hospital, Shanghai Jiao Tong University, Shanghai, China

[2] National Clinical Research Center for Eye Diseases, Shanghai, China

[3] Shanghai Key Laboratory of Ocular Fundus Diseases, Shanghai, China

[4] Shanghai Engineering Center for Visual Science and Photomedicine, Shanghai, China

[5] Shanghai Engineering Center for Precise Diagnosis and Treatment of Eye Disease, Shanghai, China

Corresponding author: Tianwei Qian
100 Haining Road, Hongkou District, Shanghai 200080, China
Tel: +86 (0) 15201955168 Fax: 021-63240090
E-mail: qtw6180@126.com

Word count: 3791

For peer review only - http://bmjopen.bmj.com/site/about/guidelines.xhtml

Abstract

Introduction Vitrectomy combined with internal limiting membrane (ILM) peeling, flap or tamponade is widely used in the treatment of macular diseases, such as macular hole (MH) and high myopia macular hole retinal detachment (HMMHRD). However, movement of the ILM to a suitable position to prevent displacement is a difficult operation. Improving visual function after surgery remains controversial. Compared to ILM, the thicker and more flexible lens capsule is easy to obtain and operate. Previous studies have confirmed the effectiveness of lens capsule flap in the treatment of MH. This study aims to evaluate the efficacy and safety of vitrectomy combined with lens capsule flap transplantation in the treatment of HMMHRD.

Methods and analysis This single-centre, single-blind, prospective, randomised clinical trial will include 78 patients with HMMHRD who will first undergo phacoemulsification and intraocular lens implantation and then vitrectomy combined with lens capsule flap transplantation (experimental group) or ILM tamponade (control group). Study participants will be randomly allocated in a 1:1 ratio to experimental and control groups. Follow-up will be conducted 1, 3, and 7 days and 1, 3, and 6 months after surgery in both groups. Necessary examinations will be performed at each follow-up visit. Measurement outcomes include postoperative situation of macular hole closure, best corrected visual acuity, macular retinal function, and macular retinal sensitivity. The primary outcome is Type I closure rate of MH 6 months after operation. Intergroup comparisons of the proportions of patients with Type I closure of MH will be performed with Fisher's exact test.

Ethics and dissemination Full ethics approval for this study was obtained from the Ethics Committee of Shanghai General Hospital, Shanghai Jiaotong University, Shanghai, China. The outcomes of the trial will be disseminated through peer-reviewed journals and at scientific conferences.

Trial registration number ChiCTR2200057836

For peer review only - http://bmjopen.bmj.com/site/about/guidelines.xhtml

Strengths and limitations of this study

- This is the first randomised controlled trial investigating the efficacy and safety of vitrectomy combined with lens capsule flap transplantation in the treatment of high myopia macular hole retinal detachment (HMMHRD).
- In addition to anatomical measurement, functional measurements, including best corrected visual acuity, macular retinal function, and macular retinal sensitivity, are also used to evaluate the postoperative efficacy.
- Based on whether the macular hole reaches 400 μm, this trial will adopt the stratified randomization method through the central random system.
- Strict quality control measures, including adequate concealment of randomised group allocations, will be implemented.
- A limitation of this trial is that the difference in preoperative lens opacity may affect the baseline visual acuity of the two groups.

For peer review only

Introduction

High myopia is a common cause of vision loss. Globally, about 163 million individuals (2.7% of the world population) suffered from high myopia in 2000. With the increasing prevalence of HM, an estimated 1 billion individuals (9.8% of the world population) will suffer from high myopia in 2050.[1] Patients with high myopia have a higher risk of ocular disorders, such as cataract and glaucoma, than those without high myopia. In the field of the fundus, high myopia is associated with a series of pathological complications, including posterior staphyloma, myopic macular schisis (MMS), preretinal membrane, chorioretinal dystrophy, macular hole (MH), and retinal detachment (RD).[2-4] In patients with high myopia, about 6% of patients will develop MH, which is about 10 times higher than in individuals without high myopia.[5] Patients with high myopia have a long ocular axis and a high risk of posterior scleral staphyloma. Continuous traction of the vitreous leads to the formation of macular holes. The liquid in the vitreous reaches beneath the retinal neuroepithelial layer through the macular holes to form subretinal fluid (SRF), which will lead to high myopia macular hole retinal detachment (HMMHRD).[6] HMMHRD can be clearly diagnosed by optical coherence tomography (OCT). Once it occurs, vision deteriorates drastically, reducing quality of life. It is one of the serious complications threatening the vision of patients with high myopia.[7 8] Possible mechanisms of HMMHRD include vitreoretinal traction caused by epiretinal membrane, residual vitreous cortex, inner limiting membrane (ILM), and retinal vascular system,[9] which makes the vertical and tangential traction generated by the epiretinal membrane and ILM,[10 11] and further weakens the adhesion between retinal neuroepithelial layer and pigment epithelial layer (RPE). The long axis reduces the extension of the retina, resulting in the relative shortening of the retina, and subsequent retinal splitting and detachment.[12]

The key to the treatment of HMMHRD is to restore the normal anatomical structure of the macular region, improve the continuous traction state of the vitreous to the retina, and induce retinal reattachment. The main surgical procedures include extrascleral surgery (scleral buckling, macular banding, and posterior scleral reinforcement), intraocular surgery (vitrectomy), and combined intraocular and extraocular surgery. Currently, vitreoretinal

For peer review only - http://bmjopen.bmj.com/site/about/guidelines.xhtml

surgery (VRS) is the mainstream surgical method for the treatment of HMMHRD. Among them, pars plana vitrectomy (PPV) can relieve the mechanical traction of the vitreous cortex on the macula, restore the anatomical structure, effectively reduce retinal oedema and facilitate fluid exchange, and promote the recovery of retinal function in the macular region. It is one of the most routinely performed operations.[13] However, the abnormalities of the anatomical structure in high myopia, such as long ocular axis, retinal choroidal atrophy at the posterior pole, posterior scleral staphyloma, and "macular white hole", lead to increased surgical difficulty, resulting in low retinal reduction and hole closure rate and high recurrence rate in patients with HMMHRD after simple vitrectomy. In addition, scar healing, retinal interlayer structure disorder, RPE atrophy, and ganglion cell damage occur in macular holes after operation, which lead to unsatisfactory visual function recovery, even if retinal anatomical reduction was achieved.[14 15] Therefore, to improve the success rate of surgery and preserve the visual function of patients as much as possible, combined macular interface surgery is needed after vitrectomy removes the traction of the vitreous to the macula. The combination of surgical methods, such as ILM peeling, ILM flap, and ILM tamponade, are currently in practice. Improving the visual function of patients based on improving the reduction rate is the key to the selection of macular interface surgery for HMMHRD.[16 17]

Although these operations can improve the healing rate of macular hole, obtaining the inner limiting membrane tissue at the edge in patients with high myopia remains challenging. The challenge is due to the problems of posterior scleral staphyloma, retinal choroidal atrophy, and difficulty in identifying the inner limiting membrane.[18 19] Moreover, because the ILM is originally soft, under the influence of the long axis of high myopia, ILM softens further under traction. Therefore, moving the ILM to a suitable position to prevent displacement is difficult.[19 20] As a result, the role of improving visual function post-surgery remains controversial. Therefore, the choice of operation mode of macular interface is particularly important.

In 2019, Grewal *et al*[21] first reported that the lens capsule was transplanted to the macular hole for the treatment of refractory macular hole. Peng *et al*[22 23] used an autologous or allogeneic lens capsule to treat refractory macular hole. The healing rate can reach 90% and

For peer review only - http://bmjopen.bmj.com/site/about/guidelines.xhtml

96%, and the visual acuity of patients can be improved. Similar to the ILM, the lens capsule is a kind of basement membrane. Although the lens capsule is thicker than the ILM, it is quite flexible. Therefore, positioning the lens capsule at a designated place in the retina is easier than the ILM. For most patients who need surgery for HMMHRD, the degree of lens turbidity reaches the surgical indication of cataract phacoemulsification. Therefore, most doctors will choose to complete the operation together with cataract surgery to easily obtain the capsule tissue. The mechanism of lens capsule transplantation in the treatment of macular hole is similar to that of ILM. The lens capsule can be used as a scaffold to promote the proliferation and migration of Müller cells in the retina and promote the healing of macular holes. The study found that the lens capsule as a stent still existed and did not become thinner after nearly 3 years of follow-up.[23] Appropriate size and reasonable placement of lens capsule graft is the key to successful surgery.[23] Mastery of certain surgical skills is necessary to fill the lens capsule at the macular hole. Moreover, lens capsule transplantation cannot only promote the healing of macular hole but also improve the vision of patients.[20 24 25] In addition, lens epithelial cells (LECs) on the anterior capsule flap can be removed using distilled water, resulting in low antigenicity.[23] In the actual operation of capsule transplantation, Chen *et al*[16 20] reported that the lens capsule is easier to process and insert MH than ILM, making it possible to vigorously promote this technology.

Based on the advantages of easy access, easy operation of lens capsule, combined with the clinical prognosis of high retinal reattachment rate and better improvement of visual function found in the capsule transplantation group in the previous study, our team plans to conduct a prospective randomized controlled trial of vitrectomy combined with lens capsule flap transplantation in the treatment of HMMHRD to explore the efficacy and safety of this operation in retinal reattachment and functional recovery after the treatment of HMMHRD.

Methods and analysis

Overview

This is a single-blind, prospective, and randomised clinical trial (RCT) being conducted at

For peer review only - http://bmjopen.bmj.com/site/about/guidelines.xhtml

Shanghai General Hospital affiliated with Shanghai Jiaotong University. The patients have a clear diagnosis of HMMHRD and require vitrectomy. Our hypothesis is that vitrectomy combined with lens capsule flap transplantation in the treatment of HMMHRD can achieve a higher proportion of retinal reattachment and better functional recovery. After comprehensive ophthalmic and physical examinations, the patients will be randomised into an experimental group and control group to receive treatment. The first participant will be enrolled in April 2022. The projected completion date is March 2025.

Objective

The study aims to evaluate the efficacy and safety of vitrectomy combined with lens capsule flap transplantation in retinal reattachment and functional recovery in patients with HMMHRD.

Trial design

The prospective, single-centre RCT will enrol 78 participants that will be randomised in a 1:1 ratio to the experimental group (vitrectomy combined with lens capsule flap transplantation) or control group (vitrectomy combined with ILM tamponade). The patient's ophthalmic and systemic medical history and first examination results at admission will be recorded during the screening visit for the trial. Vitrectomy combined with lens capsule flap transplantation or ILM tamponade will be performed by one experienced surgeon. The protocol (date: 15/03/2022, version: 1.0) was developed in accordance with the Consolidated Standards of Reporting Trials guidelines. This protocol was written in accordance with the Standard Protocol Items: Recommendations for Interventional Trials guidelines[26] and was approved by the Institutional Review Board of Shanghai General Hospital (Supplementary 1, approved ID: 2022SQ284). The trial has been registered with the Chinese Clinical Trial Registry (ChiCTR, http://www.chictr.org.cn/, No. ChiCTR2200057836). Any protocol modifications will be communicated to the participants and trial registry. Trial results will be disseminated on trial registration (clinicaltrials.gov) or through a publication. Participants have the right to withdraw their consent and discontinue participation at any time. A flowchart of the trial design is shown

For peer review only - http://bmjopen.bmj.com/site/about/guidelines.xhtml

in Figure 1.

Recruitment

Participants will be recruited by the surgeon during hospitalisation without any other form of advertisement. Interested patients will be invited to discuss the benefits and potential risks related to the vitrectomy combined with lens capsule flap transplantation. Participants meeting all the inclusion criteria will be fully informed of their responsibilities and all procedures involved in the trial. They will be asked to sign the written informed consent before enrolment. The English translation of informed consent can be seen in Supplementary 2.

Inclusion criteria

The inclusion criteria are as follows: (1) Patients ≥50 years old who were diagnosed as HMMHRD by indirect ophthalmoscopy, macular OCT, fundus photography, and B-ultrasound examination; (2) equivalent spherical dioptre ≥–6.00 D or axis length ≥ 26 mm; (3) MH as main cause of RD; (4) turbid lens without obvious calcification in the lens capsule, requiring combination with cataract surgery; (5) the range of RD within the retinal vascular arch of the posterior pole; and (6) consent to undergo surgical treatment and regular follow-up and sign relevant informed consent.

Exclusion criteria

Exclusion criteria are as follows: (1) Patients with RD with other types of holes except MH, choroidal detachment, obvious proliferative vitreoretinopathy, intraocular haemorrhage, severe cataract, glaucoma, retinal vascular inflammation or obstruction, macular degeneration, macular neovascularization, macular haemorrhage, pathological myopia, and macular atrophy; (2) patients with previous history of intraocular surgery (including PPV and intravitreal drug injection); (3) patients whose general condition cannot tolerate the operation; (4) patients who cannot cooperate with the examination and follow-up for <6 months; (5) patients unwilling to undergo concurrent vitrectomy and cataract surgery; (6) patients with

For peer review only - http://bmjopen.bmj.com/site/about/guidelines.xhtml

concomitant diseases or conditions of the target eye or whole body (including malignant hypertension; AIDS; malignant tumour; serious mental, cardiovascular, neurologic, respiratory, digestive, or other systemic disease; long-term use of hormones; and immunodeficiency after heart stenting or organ transplantation); (7) patients with diabetes with poor blood glucose control; (8) patients with poor communication skills and compliance; (9) exclusion of patient by researchers during the course of the study because patient is unsuitable to continue participation; and (10) participation in other trials.

Withdrawal criteria

Withdrawal criteria are as follows: (1) loss to follow-up; (2) withdrawal of informed consent; (3) participation in other clinical studies during the study; (4) emergence of other eye diseases during the study; (5) need of other eye treatments during the study; (6) Decision by researchers to withdraw participant due to safety concerns (7) Decision by researchers to withdraw patient due to unsuitability of treatment methods (including remedial treatment) for patient.

Randomisation and blinding

The stratified randomisation method will be adopted in allocating participants to the two groups in a 1:1 ratio using the central random system. The stratification factors are as follows: 1) macular hole size >400 μm and 2) macular hole size ≤400 μm. Except the surgeon, all researchers, including the outcome assessors, statisticians, and data analysts, will be blinded to group assignment, but providers of the intervention/control will be informed by necessity. Vitrectomy combined with lens capsule flap transplantation or ILM tamponade will be performed by one experienced surgeon. Before the trial, researchers will be fully trained in the randomisation procedure and made aware of their individual responsibilities. The successful implementation and maintenance of the randomisation and blinding method will be validated.

Interventions

For peer review only - http://bmjopen.bmj.com/site/about/guidelines.xhtml

All participants who meet the inclusion criteria of this study and do not meet any of the exclusion criteria can be included in this study. After passing the screening, the participants who provided written informed consent will be randomly assigned to two parallel trial groups in a 1:1 ratio according to the central randomization system. The patients will undergo vitrectomy combined with lens capsule flap transplantation or ILM tamponade and regular post-surgery follow-up. Participants will be blinded to the specific surgical methods. The timeline of data collection is shown in Table 1.

For peer review only

For peer review only - http://bmjopen.bmj.com/site/about/guidelines.xhtml

Table 1. Outcome measurements and data collection at each follow-up timepoint

Phase		Screen	Preoperative examination	Postoperative follow-up					
Follow-up		1	2	3	4	5	6	7	14
Time points		Within 1 week before operation	One day before operation	1 day after operation	3 days after operation	7 days after operation	1 month after operation	3 months after operation	6 months after operation
Informed consent		●							
Inclusion and exclusion criteria		●							
Withdrawal criteria			●	●	●	●	●	●	●
Medical history collection		●							
Ophthalmic examination	BCVA	●	●	●	●	●	●	●	●
	Slit lamp inspection	●	●	●	●	●	●	●	●
	IOP	●	●	●	●	●	●	●	●
	Ocular B-ultrasound	●	●	●	●	●	●	●	●
	OCT	●	●	●	●	●	●	●	●
	mfERG		●					●	●
	MP-1 microperimeter		●					●	●
	FFA & ICGA	●							●

For peer review only

For peer review only - http://bmjopen.bmj.com/site/about/guidelines.xhtml

Systemic examination	Vital signs	●	●	●	●	●	●	●	●
	Physical examination	●							●
Laboratory examination	routine blood test	●	●				●		●
	Blood biochemistry	●					●		●
	Coagulation function	●							●
	Urinalysis	●							●
	Immune and infection measurements	●				●	●		●
	Electrocardiogram	●							●
	Chest CT	●							●
Adverse events recorded		●	●	●	●	●	●	●	●

BCVA, best corrected visual acuity; IOP, intraocular pressure; OCT, optical coherence tomography; mfERG, multifocal electrophysiology; FFA, fluorescence fundus angiography; ICGA, indocyanine green angiography.

For peer review only

For peer review only - http://bmjopen.bmj.com/site/about/guidelines.xhtml

Experimental group

Patients will first undergo phacoemulsification and intraocular lens implantation. During this operation, an appropriate size of the lens capsule flap will be obtained according to the size of the macular hole and placed in sterile distilled water for at least 30 minutes. Thereafter, the 23-gauge vitrectomy through the pars plana will be performed, and the ILM will be stained with indocyanine green. After indocyanine green staining, the ILM in the macular area will be completely removed with intraocular forceps. Afterwards, the lens capsule flap will need to be inserted into the macular hole to complete gas-liquid exchange. In this process, the operator will ensure that the capsule flap is always right under the macular hole. Finally, silicone oil will be injected into the vitreous cavity.

Control group

Patients will first undergo phacoemulsification and intraocular lens implantation. Thereafter, 23-gauge vitrectomy through the pars plana will be performed. After complete vitreous removal, ILM will be stained with indocyanine green. After indocyanine green staining, the ILM of the retina in the indocyanine green staining area will be directly clamped, and the ILM ring in the area will be removed about 1.5-disc diameters away from the macular hole. Care will be taken to prevent peeling off the ILM valve at the edge of the macular hole. Thereafter, the free ILM flap will be turned over and filled into the MH. During the operation, accurate filling of ILM into the MH will be confirmed severally. After complete gas-liquid exchange, silicone oil will be injected into the vitreous cavity.

Postoperative management

All patients will be administered with anti-inflammatory and anti-infective treatment after operation. During hospitalisation, IOP will be monitored 2–3 times daily. If IOP increases, it will be reduced. In case it is not controlled, mannitol will be administered intravenously to control IOP. If necessary, anterior chamber puncture, aspiration, and drainage or glaucoma surgery will be performed.

For peer review only - http://bmjopen.bmj.com/site/about/guidelines.xhtml

Outcome measurements

Follow-up will be conducted at 1, 3, and 7 days and 1, 3, and 6 months after surgery in both groups. Necessary examinations will be performed at each follow-up visit.

Anatomical measurement

OCT will be used to assess the retina and macula before and after surgery. According to the postoperative situation of retinal reattachment and macular hole closure, the surgical result will be classified as: (1) Type I closure: the two ends of the macular hole are closely connected and close to the RPE layer without any obvious exposure of the RPE layer and subretinal fluid (SRF); (2) Type II closure: the two ends of the macular hole are not connected, and the edge of the hole is closely attached to the RPE layer with a distance between the two ends. The exposed RPE layer can be observed without any obvious SRF; (3) Unclosed: the two ends of the macular hole are not connected, and the edge of the hole is still raised and tilted away from the RPE layer. The SRF can be observed below.[27 28]

Functional measurement

Postoperative visual acuity, macular retinal function, and macular retinal sensitivity are all important functional outcomes. (1) Central vision: best corrected visual acuity (BCVA); (2) macular retinal function: multifocal electrophysiology (mfERG) collects the retinal amplitude information caused by specific stimulation, forms a three-dimensional topographic map, and collects P1 wave response density data for intuitive evaluation of macular retinal function; (3) Macular retinal sensitivity: MP-1 microperimetry is used to examine the micro visual field of the macula.

Primary outcome

The primary outcome is Type I closure rate of MH 6 months after operation.

For peer review only - http://bmjopen.bmj.com/site/about/guidelines.xhtml

Secondary outcomes

The following indicators will be used to comprehensively assess anatomical outcome: (1) Type I closure rate of macular hole 3 months after operation; (2) type II closure rate of macular hole 6 months after operation; and (3) type II closure rate of macular hole 3 months after operation. Functional outcomes will be assessed as follows: (1) Changes in BCVA 3 and 6 months after operation compared to baseline; (2) changes in P1 wave response density in the macular region 3 and 6 months after operation compared to baseline; and (3) changes in macular retinal sensitivity 3 and 6 months after operation compared to baseline.

Furthermore, the number of adverse events and reactions, abnormality of ophthalmic examination, abnormal systemic symptoms, and laboratory results will be recorded at each follow-up visit.

Data collection and safety monitoring board

Basic demographic information and data on history of ocular diseases will be collected pre-intervention. To ensure the integrity of the RCT and protect the rights and health of the participants, a Data and Safety Monitoring Board will be constituted. As an independent advisory group, it will ensure the scientific integrity and ethical standards of the RCT and be responsible for data evaluation during the study period. The Data and Safety Monitoring Board will be developed in accordance with the Operational Guidelines for the Establishment and Functioning of Data and Safety Monitoring Boards of the WHO. Researchers will be trained to collect good quality data, promote participant retention, and complete follow-up. Data analysts will be trained on data entry, coding, security, and storage. Statisticians will provide training on data assessment and statistical analysis.

Safety and combined operation-related adverse events

Possible phacoemulsification and intraocular lens implantation combined with vitrectomy complications include intraocular haemorrhage, suprachoroidal haemorrhage, endophthalmitis, uveitis, intraocular lens dislocation, and corneal endothelial decompensation. Lens capsule flap

For peer review only

transplantation may increase the risk of rejection, although the capsule flap will be immersed in sterile distilled water for 30 minutes. However, to date, no adverse reactions caused by lens capsule flap transplantation for the treatment of MH have been reported. During the trial, we will record and analyse any surgery-related adverse events (including the type and number) in each group and possible causes. Patients will receive appropriate intervention for any adverse events that occur during the treatment and follow-up phases. Severe adverse events will be reported immediately to the primary investigator and affected participants will be withdrawn from the study.

Patients and public involvement

Neither the patients nor the public will be involved in the design of the study; nor will they be involved in its conduct (including outcome measurements). No attempt will be made to assess the burden of the intervention on the patients by themselves. Results excluding personal data will be made available on request.

Sample size

According to previous retrospective data generated by our study group, the Type I closure rate in the experimental group was 91.7%, whereas the rate in the control group was 64.3%. An a priori power analysis conducted using a two-tailed test (power = 0.8) at the 5% significance level indicated that 31 participants are required in each group. Considering the attrition rate of 20%, the sample size in each group was determined as 39. With the 1:1 allocation, 78 participants will be recruited.

Statistical analysis

SPSS (version 22.0; SPSS, Inc., Chicago, IL, USA) and GraphPad Prism (version 8, GraphPad software Inc., San Diego, CA, USA) will be used for data processing and analyses. Categorical variables will be expressed as numbers and frequencies, and between-group differences will be evaluated with Fisher's exact test. Continuous variables will be expressed as the mean ±

For peer review only - http://bmjopen.bmj.com/site/about/guidelines.xhtml

standard deviation (SD). The Kolmogorov-Smirnov test will be used to determine whether continuous variables are normally distributed. The student's t test will be used for normally distributed data, and the Wilcoxon signed-rank test will be used for non-normally distributed data. All statistical tests will be two-sided with $P < 0.05$ considered statistically significant.

Ethics and dissemination

This RCT has been designed in accordance with the principles of the Declaration of Helsinki. The trial protocol has been approved by the Shanghai General Hospital Institutional Review Board (Approval ID: 2022SQ284). It is registered on the primary registry of the WHO registry network (Chinese Clinical Trial Registry). Signed consent will be obtained from each patient after they have been informed of the study procedures, possible risks, and their right to withdraw from the trial. Neither the patients nor the public are involved in the development of the research question or study design. The results of the trial without personal data will be directly communicated to participants and to the public through peer-reviewed publications and conference presentations. Standard and on-site screening are being conducted by an experienced retinal surgeon. Anaesthesiologists will monitor the whole surgical process.

Discussion

Although the retinal changes in the macula of most patients with MH are slow and may not change much after several years, once MHHMRD occurs, the visual function is often severely damaged with a poor prognosis. Therefore, the treatment of HM-related MHRD is not only a medical problem but also a public health problem.

In addition, the size of the macular hole is closely related to anatomical reduction, and the minimum diameter of the hole is the most predictive parameter of MH closure. According to the anatomical classification system of OCT, the distance between the two points at the narrowest place on both sides of the hole at the maximum diameter of MH is measured as the size of the macular hole,[29] which is divided into small (≤250 μm), medium (>250 μm and ≤ 400 μm) and large (> 400 μm). Freeman et al.[30] found that the MH with diameter ≤400 μm is

For peer review only - http://bmjopen.bmj.com/site/about/guidelines.xhtml

related to better functional results, which provides a theoretical basis for the stratified randomization in the design of this study. The mechanism of lens capsule flap transplantation in the treatment of macular hole is similar to that of ILM. The lens capsule flap can be used as a scaffold to promote the proliferation and migration of Müller cells in the retina to promote the healing of MH.

This article describes the design and protocol of a study that aims to evaluate the efficacy and safety of vitrectomy combined with lens capsule flap transplantation in the treatment of HMMHRD. The results of this RCT will reveal whether vitrectomy combined with lens capsule flap transplantation has a significant effect on MH closure and visual recovery in patients with HMMHRD. If the results of this trial are significant, a large-scale multi-centre clinical trial can be designed.

Trial status

This study is currently in the preparatory stage. The first participant will be enrolled in April 2023. The projected completion date is March 2026.

Acknowledgements

The trial will be conducted at Shanghai General Hospital. The authors thank the patients and their family members, as well as the nurses, pharmacy staff, fellows, statisticians, and clinicians for making this study possible.

Contributors

TQ, QG, LY, YF, WW, and XX participated in trial design. TQ, QG, LX, YF, and WW designed the data analysis plan. LY, XW, HZ, and WW collected the information needed to conduct this trial. QG, WW, and TQ wrote the first draft of the manuscript. All authors reviewed and revised the manuscript and gave final approval for the publication of this study protocol.

For peer review only

Funding

This study was supported by Special Project for Clinical Research in Health Industry of Shanghai Health Commission (No. 20214Y0045).

Competing interests

None declared.

Ethics approval

The study protocol has been approved by the Shanghai General Hospital Institutional Review Board (Approved ID: 2022SQ284).

Provenance and peer review

Not commissioned; externally peer reviewed.

Data sharing statement

The submitted manuscript is a study protocol without primary data. Further information can be obtained from the corresponding author.

For peer review only

For peer review only - http://bmjopen.bmj.com/site/about/guidelines.xhtml

References

1. Holden BA, Fricke TR, Wilson DA, et al. Global Prevalence of Myopia and High Myopia and Temporal Trends from 2000 through 2050. *Ophthalmology* 2016;123(5):1036-42. doi: 10.1016/j.ophtha.2016.01.006 [published Online First: 2016/02/15]
2. Henaine-Berra A, Zand-Hadas IM, Fromow-Guerra J, et al. Prevalence of macular anatomic abnormalities in high myopia. *Ophthalmic surgery, lasers & imaging retina* 2013;44(2):140-4. doi: 10.3928/23258160-20130219-01 [published Online First: 2013/02/27]
3. Rino F, Elena Z, Ivan M, et al. Lamellar macular hole in high myopic eyes with posterior staphyloma: morphological and functional characteristics. *Graefe's archive for clinical and experimental ophthalmology = Albrecht von Graefes Archiv fur klinische und experimentelle Ophthalmologie* 2016;254(11):2141-50. doi: 10.1007/s00417-016-3371-5 [published Online First: 2016/10/27]
4. Chang L, Pan CW, Ohno-Matsui K, et al. Myopia-related fundus changes in Singapore adults with high myopia. *American journal of ophthalmology* 2013;155(6):991-99.e1. doi: 10.1016/j.ajo.2013.01.016 [published Online First: 2013/03/19]
5. Margheria RR, Schepens CL. Macular breaks. 1. Diagnosis, etiology, and observations. *American journal of ophthalmology* 1972;74(2):219-32. [published Online First: 1972/08/01]
6. Liu B, Chen S, Li Y, et al. Comparison of macular buckling and vitrectomy for the treatment of macular schisis and associated macular detachment in high myopia: a randomized clinical trial. *Acta ophthalmologica* 2020;98(3):e266-e72. doi: 10.1111/aos.14260 [published Online First: 2019/11/19]
7. Lichtwitz O, Boissonnot M, Mercié M, et al. Prevalence of macular complications associated with high myopia by multimodal imaging. *Journal francais d'ophtalmologie* 2016;39(4):355-63. doi: 10.1016/j.jfo.2015.11.005 [published Online First: 2016/03/27]
8. Haarman AEG, Tedja MS, Brussee C, et al. Prevalence of Myopic Macular Features in Dutch Individuals of European Ancestry With High Myopia. *JAMA ophthalmology* 2022;140(2):115-23. doi: 10.1001/jamaophthalmol.2021.5346 [published Online First: 2021/12/17]
9. Kamal-Salah R, Morillo-Sanchez MJ, Rius-Diaz F, et al. Relationship between paravascular abnormalities and foveoschisis in highly myopic patients. *Eye (London, England)* 2015;29(2):280-5. doi: 10.1038/eye.2014.255 [published Online First: 2014/11/02]
10. Gao X, Guo J, Meng X, et al. A meta-analysis of vitrectomy with or without internal limiting membrane peeling for macular hole retinal detachment in the highly myopic eyes. *BMC ophthalmology* 2016;16:87. doi: 10.1186/s12886-016-0266-5 [published Online First: 2016/06/15]
11. Gandorfer A, Scheler R, Haritoglou C, et al. Pathology of the macular hole rim in flat-mounted internal limiting membrane specimens. *Retina (Philadelphia, Pa)*

For peer review only

For peer review only - http://bmjopen.bmj.com/site/about/guidelines.xhtml

2009;29(8):1097-105. doi: 10.1097/IAE.0b013e3181aa8fb1 [published Online First: 2009/09/08]
12. Morita H, Ideta H, Ito K, et al. Causative factors of retinal detachment in macular holes. *Retina (Philadelphia, Pa)* 1991;11(3):281-4. doi: 10.1097/00006982-199111030-00002 [published Online First: 1991/01/01]
13. Peng KL, Kung YH, Hsu CM, et al. Surgical outcomes of centripetal non-fovea-sparing internal limiting membrane peeling for myopic foveoschisis with and without foveal detachment: a follow-up of at least 3 years. *The British journal of ophthalmology* 2020;104(9):1266-70. doi: 10.1136/bjophthalmol-2019-314972 [published Online First: 2019/12/14]
14. Yamashiro K, Kinoshita-Nakano E, Ota T, et al. Floating flap of internal limiting membrane in myopic macular hole surgery. *Graefe's archive for clinical and experimental ophthalmology = Albrecht von Graefes Archiv fur klinische und experimentelle Ophthalmologie* 2018;256(4):693-98. doi: 10.1007/s00417-018-3936-6 [published Online First: 2018/02/18]
15. Ohno-Matsui K, Jonas JB. Posterior staphyloma in pathologic myopia. *Progress in retinal and eye research* 2019;70:99-109. doi: 10.1016/j.preteyeres.2018.12.001 [published Online First: 2018/12/12]
16. Chen SN, Hsieh YT, Yang CM. Multiple Free Internal Limiting Membrane Flap Insertion in the Treatment of Macular Hole-Associated Retinal Detachment in High Myopia. *Ophthalmologica Journal international d'ophtalmologie International journal of ophthalmology Zeitschrift fur Augenheilkunde* 2018;240(3):143-49. doi: 10.1159/000487337 [published Online First: 2018/06/07]
17. Michalewska Z, Michalewski J, Dulczewska-Cichecka K, et al. Inverted internal limiting membrane flap technique for surgical repair of myopic macular holes. *Retina (Philadelphia, Pa)* 2014;34(4):664-9. doi: 10.1097/iae.0000000000000042 [published Online First: 2013/11/23]
18. Morizane Y, Shiraga F, Kimura S, et al. Autologous transplantation of the internal limiting membrane for refractory macular holes. *American journal of ophthalmology* 2014;157(4):861-69.e1. doi: 10.1016/j.ajo.2013.12.028 [published Online First: 2014/01/15]
19. Lai CC, Chen YP, Wang NK, et al. Vitrectomy with Internal Limiting Membrane Repositioning and Autologous Blood for Macular Hole Retinal Detachment in Highly Myopic Eyes. *Ophthalmology* 2015;122(9):1889-98. doi: 10.1016/j.ophtha.2015.05.040 [published Online First: 2015/07/06]
20. Chen SN, Yang CM. LENS CAPSULAR FLAP TRANSPLANTATION IN THE MANAGEMENT OF REFRACTORY MACULAR HOLE FROM MULTIPLE ETIOLOGIES. *Retina (Philadelphia, Pa)* 2016;36(1):163-70. doi: 10.1097/iae.0000000000000674 [published Online First: 2015/07/23]
21. Grewal DS, Charles S, Parolini B, et al. Autologous Retinal Transplant for Refractory Macular Holes: Multicenter International Collaborative Study Group. *Ophthalmology*

For peer review only - http://bmjopen.bmj.com/site/about/guidelines.xhtml

2019;126(10):1399-408. doi: 10.1016/j.ophtha.2019.01.027 [published Online First: 2019/02/04]
22. Peng J, Chen C, Jin H, et al. AUTOLOGOUS LENS CAPSULAR FLAP TRANSPLANTATION COMBINED WITH AUTOLOGOUS BLOOD APPLICATION IN THE MANAGEMENT OF REFRACTORY MACULAR HOLE. *Retina (Philadelphia, Pa)* 2018;38(11):2177-83. doi: 10.1097/iae.0000000000001830 [published Online First: 2017/10/19]
23. Peng J, Chen C, Zhang H, et al. LONG-TERM SURGICAL OUTCOMES OF LENS CAPSULAR FLAP TRANSPLANTATION IN THE MANAGEMENT OF REFRACTORY MACULAR HOLE. *Retina (Philadelphia, Pa)* 2021;41(4):726-34. doi: 10.1097/iae.0000000000002922 [published Online First: 2020/08/01]
24. Gosala S, Manchikanty SM. Inverted Internal Limiting Membrane Insertion for Macular Hole-Associated Retinal Detachment in High Myopia. *American journal of ophthalmology* 2016;166:210-11. doi: 10.1016/j.ajo.2016.04.006 [published Online First: 2016/05/14]
25. Peng J, Chen C, Zhang L, et al. LENS CAPSULAR FLAP TRANSPLANTATION AS PRIMARY TREATMENT FOR CLOSURE OF LARGE MACULAR HOLES. *Retina (Philadelphia, Pa)* 2022;42(2):306-12. doi: 10.1097/iae.0000000000003315 [published Online First: 2022/01/21]
26. Chan AW, Tetzlaff JM, Gøtzsche PC, et al. SPIRIT 2013 explanation and elaboration: guidance for protocols of clinical trials. *BMJ (Clinical research ed)* 2013;346:e7586. doi: 10.1136/bmj.e7586 [published Online First: 2013/01/11]
27. Kang SW, Ahn K, Ham DI. Types of macular hole closure and their clinical implications. *The British journal of ophthalmology* 2003;87(8):1015-9. doi: 10.1136/bjo.87.8.1015 [published Online First: 2003/07/26]
28. Imai M, Iijima H, Gotoh T, et al. Optical coherence tomography of successfully repaired idiopathic macular holes. *American journal of ophthalmology* 1999;128(5):621-7. doi: 10.1016/s0002-9394(99)00200-7 [published Online First: 1999/11/30]
29. Cheng Y, Zou H, Zhang X, et al. Pars plana vitrectomy with air tamponade for the treatment of medium-large macular holes. *Clinical & experimental optometry* 2020;103(6):843-46. doi: 10.1111/cxo.13145 [published Online First: 2020/09/19]
30. Freeman WR, Azen SP, Kim JW, et al. Vitrectomy for the treatment of full-thickness stage 3 or 4 macular holes. Results of a multicentered randomized clinical trial. The Vitrectomy for Treatment of Macular Hole Study Group. *Archives of ophthalmology (Chicago, Ill : 1960)* 1997;115(1):11-21. doi: 10.1001/archopht.1997.01100150013002 [published Online First: 1997/01/01]

For peer review only - http://bmjopen.bmj.com/site/about/guidelines.xhtml

Figure Legend

Figure 1 Flowchart of the trial design.

For peer review only

For peer review only - http://bmjopen.bmj.com/site/about/guidelines.xhtml

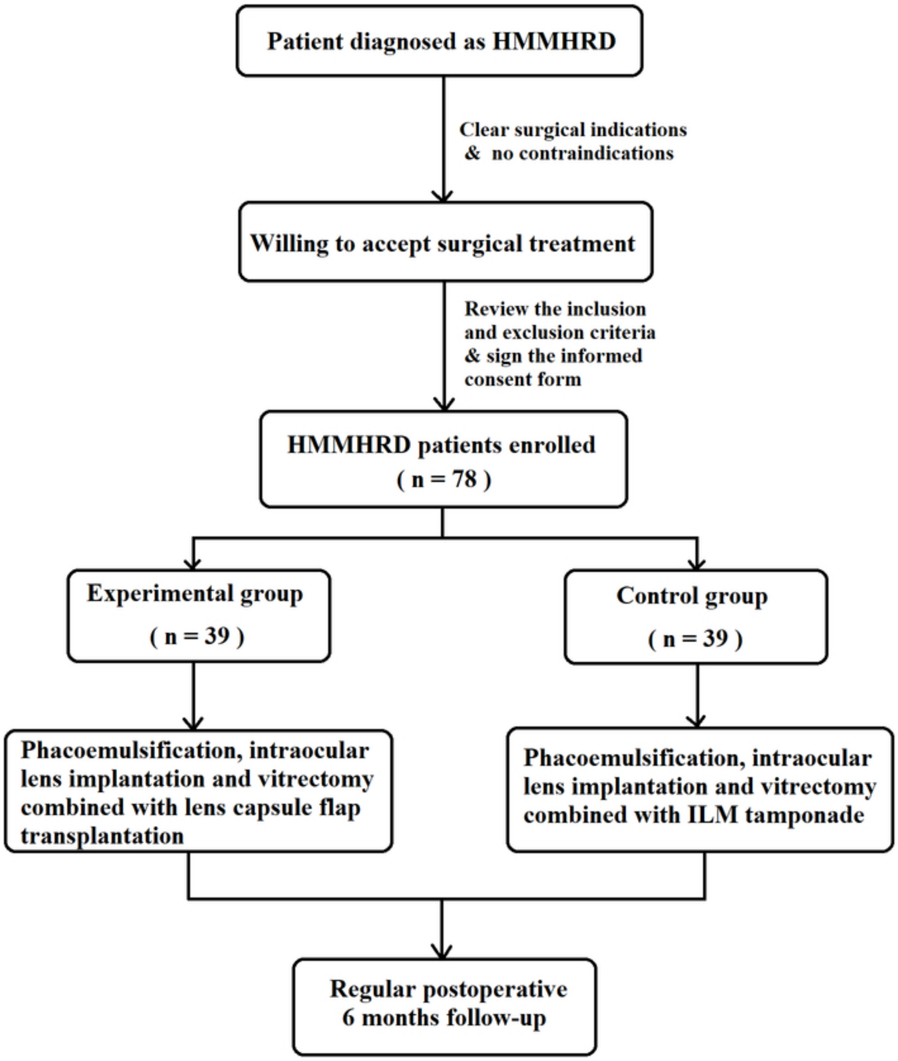

Flowchart of the trial design

66x73mm (300 x 300 DPI)

Institutional Review Board of Shanghai General Hospital

Approval Letter of Shanghai General Hospital Institutional Review Board

**Approval No:** 2022SQ284	**Review date:** Mar 16, 2022	**Project No:** 2022SQ284

**Study Protocol**	A prospective randomized controlled trial of vitrectomy combined with lens capsule flap transplantation in the treatment of high myopia macular hole retinal detachment		
**Project source**	Initiated by researchers		
**Principal investigator**	Tianwei Qian		
**Research Center**	Shanghai General Hospital	**Department**	Ophthalmology

Review documents (including version number)

(1) Study protocol

(2) Resume of principal investigator

(3) Informed consent

1. Review method

Rapid review

2. Results

Agree

3. The study will be subject to ongoing review by the ethics committee?

■ **Yes** ☐**No**

The censorship frequency is once every 12 months from the date of approval. The ethics committee has the right to change the frequency of continuous review according to the actual situation.

Institutional Review Board

Date: Mar 16, 2022

Address: No.100 Haining Road, Shanghai **Official E-mail:** shiyilunli@sina.com

Informed Consent Notification Page

Trial: A prospective randomized controlled trial of vitrectomy combined with lens capsule flap transplantation in the treatment of high myopia macular hole retinal detachment

Sponsor：Shanghai General Hospital

We invite you to participate in a study: A prospective randomized controlled trial of vitrectomy combined with lens capsule flap transplantation in the treatment of high myopia macular hole retinal detachment. Before you decide to participate in this trial, please read this informed consent carefully. If you have any questions you don't understand, you can ask the researcher in charge of the trial or members of the trial working group to explain any terms or materials you don't know.

1. Research background and purpose

1.1 Research background

Vitrectomy combined with internal limiting membrane (ILM) peeling, flap or tamponade is widely used in the treatment of macular diseases, such as macular hole (MH) and high myopia macular hole retinal detachment (HMMHRD). However, movement of the ILM to a suitable position to prevent displacement is a difficult operation. Improving visual function after surgery remains controversial. Compared to ILM, the thicker and more flexible lens capsule is easy to obtain and operate. Previous studies have confirmed the effectiveness of lens capsule flap in the treatment of MH.

1.2 Research purpose

This study aims to evaluate the efficacy and safety of vitrectomy combined with lens capsule flap transplantation in the treatment of HMMHRD.

2. Methods

2.1 Who can take part in the trial?

The inclusion criteria are as follows:

For peer review only - http://bmjopen.bmj.com/site/about/guidelines.xhtml

1） Patients ≥ 50 years old who were diagnosed as HMMHRD by indirect ophthalmoscopy, macular OCT, fundus photography, and B-ultrasound examination;

2） equivalent spherical dioptre ≥ − 6.00 D or axis length ≥ 26 mm;

3） MH as main cause of RD;

4） turbid lens without obvious calcification in the lens capsule, requiring combination with cataract surgery;

5） the range of RD within the retinal vascular arch of the posterior pole;

6） consent to undergo surgical treatment and regular follow-up and sign relevant informed consent.

2.2 Who can't take part in the experiment?

1） patients with RD with other types of holes except MH, choroidal detachment, obvious proliferative vitreoretinopathy, intraocular haemorrhage, severe cataract, glaucoma, retinal vascular inflammation or obstruction, macular degeneration, macular neovascularization, macular haemorrhage, pathological myopia, and macular atrophy;

2） patients with previous history of intraocular surgery (including PPV and intravitreal drug injection)

3） patients whose general condition cannot tolerate the operation;

4） patients who cannot cooperate with the examination and follow-up for <6 months;

5） patients unwilling to undergo concurrent vitrectomy and cataract surgery;

6） patients with concomitant diseases or conditions of the target eye or whole body (including malignant hypertension; AIDS; malignant tumour; serious mental, cardiovascular, neurologic, respiratory, digestive, or other systemic disease; long-term use of hormones; and immunodeficiency after heart stenting or organ transplantation);

7） patients with diabetes with poor blood glucose control;

8） patients with poor communication skills and compliance;

9） exclusion of patient by researchers during the course of the study because patient is unsuitable to continue participation;

10） participation in other trials.

For peer review only - http://bmjopen.bmj.com/site/about/guidelines.xhtml

2.3 Trial design and sample size

The prospective, single-centre RCT will enrol 78 participants that will be randomised in a 1:1 ratio to the experimental group (vitrectomy combined with lens capsule flap transplantation) or control group (vitrectomy combined with ILM tamponade).

2.4 Interventions

2.4.1 Experimental group

Patients will first undergo phacoemulsification and intraocular lens implantation. During this operation, an appropriate size of the lens capsule flap will be obtained according to the size of the macular hole and placed in sterile distilled water for at least 30 minutes. Thereafter, the 23-gauge vitrectomy through the pars plana will be performed, and the ILM will be stained with indocyanine green. After indocyanine green staining, the ILM in the macular area will be completely removed with intraocular forceps. Afterwards, the lens capsule flap will need to be inserted into the macular hole to complete gas-liquid exchange. In this process, the operator will ensure that the capsule flap is always right under the macular hole. Finally, silicone oil will be injected into the vitreous cavity.

2.4.2 Control group

Patients will first undergo phacoemulsification and intraocular lens implantation. Thereafter, 23-gauge vitrectomy through the pars plana will be performed. After complete vitreous removal, ILM will be stained with indocyanine green. After indocyanine green staining, the ILM of the retina in the indocyanine green staining area will be directly clamped, and the ILM ring in the area will be removed about 1.5-disc diameters away from the macular hole. Care will be taken to prevent peeling off the ILM valve at the edge of the macular hole. Thereafter, the free ILM flap will be turned over and filled into the MH. During the operation, accurate filling of ILM into the MH will be confirmed severally. After complete gas-liquid exchange, silicone oil will be injected into the vitreous cavity.

2.5 Research procedures

After fully informed and signed the informed consent, the subjects could enter the screening period; In the screening period, the evaluation researchers evaluated whether they were included or not according to the inclusion/exclusion criteria. Only the qualified subjects can enter the study treatment period. The research doctor needs your cooperation in the implementation of relevant examination items to determine whether you meet the inclusion and exclusion criteria. These

For peer review only - http://bmjopen.bmj.com/site/about/guidelines.xhtml

examinations are routine examinations for clinical diagnosis and treatment of this disease.

All participants who meet the inclusion criteria of this study and do not meet any of the exclusion criteria can be included in this study. After passing the screening, the participants who provided written informed consent will be randomly assigned to two parallel trial groups in a 1:1 ratio according to the central randomization system. The patients will undergo vitrectomy combined with lens capsule flap transplantation or ILM tamponade and regular post-surgery follow-up. Participants will be blinded to the specific surgical methods.

3. Research risks and benefits

3.1 Research risks

When your health condition is harmed by participating in this trial, the research doctor will take necessary medical measures. If you have any side effects or discomfort during the trial, it is essential that you report to the study doctor immediately. You may be given other drugs to control side effects. When participating in this study, you will need to provide some personal information, and we will take all necessary measures to ensure the confidentiality of the information.

3.2 Research benefits

Your participation may be helpful to further explore the efficacy and safety of vitrectomy combined with lens capsule flap transplantation in the treatment of HMMHRD.

4. Your rights

You have the right to decide whether to participate in the experiment. If you can't make a decision immediately, you have enough time to consider it. If necessary, you can discuss with relatives, friends and other people you trust before making a decision. If you decide not to participate in this trial, your relationship with the researcher and the sponsor will not be affected, you will not be discriminated against or retaliated, and your treatment and rights will not be affected. If you decide to participate in this trial, we hope you can complete the trial without special reasons, but you have the right to withdraw at any time during the trial. If you decide to quit, please let the researcher know in time.

During the experiment, you can know the information related to you at any time. If you have any questions about this study, or if you feel unwell during the treatment, please contact the attending doctor. Contact doctor: Tianwei Qian 15201955168. And you have the right to consult about your rights or related risks.

For peer review only - http://bmjopen.bmj.com/site/about/guidelines.xhtml

5. Privacy and confidentiality

The personal information (such as name, gender, contact information, questionnaire, etc.) provided by you to the researcher may be known to the following persons or units in addition to the needs of normal research:

- Staff (inspectors, inspectors, etc.) of research funding institutions related to this experiment；
- State and local food and Drug Administration and other administrative agencies.

However, no one is allowed to disclose your personal information to others or other organizations without your permission. Except for researchers and administrative organizations, no other person or organization has the right to contact you about this experiment or provide you with information about this experiment directly.

The results of this experiment may be published in the form of academic papers, but your personal information will not appear in any publicly published documents.

6. Others

6.1 In case of the following situations, the researcher may withdraw you from the trial without your consent for your health:：

- If you continue to participate in this trial, your risk may outweigh your benefit;
- You did not follow the guidance of the researcher and did not participate in the trial according to the research plan;
- Early termination of trial.

6.2 This informed consent is in duplicate, one for the researcher and one for you.

7. Compensation for injury caused by trial

If your injury is directly caused by participating in this trial, you do not have to pay the medical expenses for treatment, which will be borne by the researcher.

For peer review only

For peer review only - http://bmjopen.bmj.com/site/about/guidelines.xhtml

Informed Consent Page

Consent statement:

1. I have read the instructions to the subjects carefully and understood the relevant background of this trial. The researchers have explained the characteristics of the study and possible adverse reactions to me in detail, and answered my questions.

2. I know that if I refuse to participate in this trial, my treatment and rights will not be affected. After understanding all the contents of the instructions to the subjects and having fully considered them, I voluntarily participate in this trial.

3. I am willing to follow the instructions of the researchers and participate in the trial according to the research protocol. During the experiment, I have the right to withdraw at any time, but before I withdraw, I need to inform the researchers in time.

4. During the trial, if there are any discomfort symptoms, I will tell the researchers in time.

Signature of Subject:

/ /

Name (regular script) Signature Date

Signature of Researcher:

/ /

Name (regular script) Signature Date

Signature of agent/Guardian (if any):

The reason why the subject can't sign this page:

The relationship between the agent/guardian and the subject:

/ /

Name (regular script) Signature Date

For peer review only - http://bmjopen.bmj.com/site/about/guidelines.xhtml

Reporting checklist for protocol of a clinical trial.

Based on the SPIRIT guidelines.

Instructions to authors

Complete this checklist by entering the page numbers from your manuscript where readers will find each of the items listed below.

Your article may not currently address all the items on the checklist. Please modify your text to include the missing information. If you are certain that an item does not apply, please write "n/a" and provide a short explanation.

Upload your completed checklist as an extra file when you submit to a journal.

In your methods section, say that you used the SPIRITreporting guidelines, and cite them as:

Chan A-W, Tetzlaff JM, Gøtzsche PC, Altman DG, Mann H, Berlin J, Dickersin K, Hróbjartsson A, Schulz KF, Parulekar WR, Krleža-Jerić K, Laupacis A, Moher D. SPIRIT 2013 Explanation and Elaboration: Guidance for protocols of clinical trials. BMJ. 2013;346:e7586

		Reporting Item	Page Number
**Administrative information**			
Title	#1	Descriptive title identifying the study design, population, interventions, and, if applicable, trial acronym	P1
Trial registration	#2a	Trial identifier and registry name. If not yet	P2

For peer review only

		registered, name of intended registry	
Trial registration: data set	#2b	All items from the World Health Organization Trial Registration Data Set	P2
Protocol version	#3	Date and version identifier	P7
Funding	#4	Sources and types of financial, material, and other support	P19
Roles and responsibilities: contributorship	#5a	Names, affiliations, and roles of protocol contributors	P18
Roles and responsibilities: sponsor contact information	#5b	Name and contact information for the trial sponsor	P1
Roles and responsibilities: sponsor and funder	#5c	Role of study sponsor and funders, if any, in study design; collection, management, analysis, and interpretation of data; writing of the report; and the decision to submit the report for publication, including whether they will have ultimate authority over any of these activities	P18
Roles and responsibilities: committees	#5d	Composition, roles, and responsibilities of the coordinating centre, steering committee, endpoint adjudication committee, data management team, and other individuals or groups overseeing the trial, if	P15, P18

For peer review only

		applicable (see Item 21a for data monitoring committee)	
**Introduction**			
Background and rationale	#6a	Description of research question and justification for undertaking the trial, including summary of relevant studies (published and unpublished) examining benefits and harms for each intervention	P4-P6
Background and rationale: choice of comparators	#6b	Explanation for choice of comparators	P4-P6
Objectives	#7	Specific objectives or hypotheses	P7
Trial design	#8	Description of trial design including type of trial (eg, parallel group, crossover, factorial, single group), allocation ratio, and framework (eg, superiority, equivalence, non-inferiority, exploratory)	P7
**Methods: Participants, interventions, and outcomes**			
Study setting	#9	Description of study settings (eg, community clinic, academic hospital) and list of countries where data will be collected. Reference to where list of study sites can be obtained	P7

For peer review only

Eligibility criteria	#10	Inclusion and exclusion criteria for participants. If applicable, eligibility criteria for study centres and individuals who will perform the interventions (eg, surgeons, psychotherapists)	P8-P9
Interventions: description	#11a	Interventions for each group with sufficient detail to allow replication, including how and when they will be administered	P9-P10
Interventions: modifications	#11b	Criteria for discontinuing or modifying allocated interventions for a given trial participant (eg, drug dose change in response to harms, participant request, or improving / worsening disease)	P10-P12
Interventions: adherance	#11c	Strategies to improve adherence to intervention protocols, and any procedures for monitoring adherence (eg, drug tablet return; laboratory tests)	P10-P13
Interventions: concomitant care	#11d	Relevant concomitant care and interventions that are permitted or prohibited during the trial	P10-P13
Outcomes	#12	Primary, secondary, and other outcomes, including the specific measurement variable (eg, systolic blood pressure), analysis metric (eg, change from baseline, final value, time to event), method of aggregation (eg, median, proportion), and time point for each outcome. Explanation of the clinical relevance of chosen efficacy and harm outcomes is strongly recommended	P14-P15

For peer review only

Participant timeline	#13	Time schedule of enrolment, interventions (including any run-ins and washouts), assessments, and visits for participants. A schematic diagram is highly recommended (see Figure)	P7, P10-P12
Sample size	#14	Estimated number of participants needed to achieve study objectives and how it was determined, including clinical and statistical assumptions supporting any sample size calculations	P16
Recruitment	#15	Strategies for achieving adequate participant enrolment to reach target sample size	P8-P9
**Methods: Assignment of interventions (for controlled trials)**			
Allocation: sequence generation	#16a	Method of generating the allocation sequence (eg, computer-generated random numbers), and list of any factors for stratification. To reduce predictability of a random sequence, details of any planned restriction (eg, blocking) should be provided in a separate document that is unavailable to those who enrol participants or assign interventions	P9
Allocation concealment mechanism	#16b	Mechanism of implementing the allocation sequence (eg, central telephone; sequentially numbered, opaque, sealed envelopes), describing any steps to	P9-P10

For peer review only

		conceal the sequence until interventions are assigned	
Allocation: implementation	#16c	Who will generate the allocation sequence, who will enrol participants, and who will assign participants to interventions	P15-P16
Blinding (masking)	#17a	Who will be blinded after assignment to interventions (eg, trial participants, care providers, outcome assessors, data analysts), and how	P15-P16
Blinding (masking): emergency unblinding	#17b	If blinded, circumstances under which unblinding is permissible, and procedure for revealing a participant's allocated intervention during the trial	P15-P16
**Methods: Data collection, management, and analysis**			
Data collection plan	#18a	Plans for assessment and collection of outcome, baseline, and other trial data, including any related processes to promote data quality (eg, duplicate measurements, training of assessors) and a description of study instruments (eg, questionnaires, laboratory tests) along with their reliability and validity, if known. Reference to where data collection forms can be found, if not in the protocol	P15-P16
Data collection plan:	#18b	Plans to promote participant retention and complete	P15-P16

For peer review only

retention		follow-up, including list of any outcome data to be collected for participants who discontinue or deviate from intervention protocols	
Data management	#19	Plans for data entry, coding, security, and storage, including any related processes to promote data quality (eg, double data entry; range checks for data values). Reference to where details of data management procedures can be found, if not in the protocol	P15-P16
Statistics: outcomes	#20a	Statistical methods for analysing primary and secondary outcomes. Reference to where other details of the statistical analysis plan can be found, if not in the protocol	P14-P17
Statistics: additional analyses	#20b	Methods for any additional analyses (eg, subgroup and adjusted analyses)	P14-P17
Statistics: analysis population and missing data	#20c	Definition of analysis population relating to protocol non-adherence (eg, as randomised analysis), and any statistical methods to handle missing data (eg, multiple imputation)	P16-P17
**Methods: Monitoring**			
Data monitoring: formal committee	#21a	Composition of data monitoring committee (DMC); summary of its role and reporting structure; statement of whether it is independent from the sponsor and competing interests; and reference to	P15

For peer review only

		where further details about its charter can be found, if not in the protocol. Alternatively, an explanation of why a DMC is not needed	
Data monitoring: interim analysis	#21b	Description of any interim analyses and stopping guidelines, including who will have access to these interim results and make the final decision to terminate the trial	P15
Harms	#22	Plans for collecting, assessing, reporting, and managing solicited and spontaneously reported adverse events and other unintended effects of trial interventions or trial conduct	P15-P16
Auditing	#23	Frequency and procedures for auditing trial conduct, if any, and whether the process will be independent from investigators and the sponsor	P15-P16
**Ethics and dissemination**			
Research ethics approval	#24	Plans for seeking research ethics committee / institutional review board (REC / IRB) approval	P17
Protocol amendments	#25	Plans for communicating important protocol modifications (eg, changes to eligibility criteria, outcomes, analyses) to relevant parties (eg, investigators, REC / IRBs, trial participants, trial registries, journals, regulators)	P17

For peer review only

Consent or assent	#26a	Who will obtain informed consent or assent from potential trial participants or authorised surrogates, and how (see Item 32)	P17
Consent or assent: ancillary studies	#26b	Additional consent provisions for collection and use of participant data and biological specimens in ancillary studies, if applicable	P17
Confidentiality	#27	How personal information about potential and enrolled participants will be collected, shared, and maintained in order to protect confidentiality before, during, and after the trial	P17
Declaration of interests	#28	Financial and other competing interests for principal investigators for the overall trial and each study site	P18-P19
Data access	#29	Statement of who will have access to the final trial dataset, and disclosure of contractual agreements that limit such access for investigators	P19
Ancillary and post trial care	#30	Provisions, if any, for ancillary and post-trial care, and for compensation to those who suffer harm from trial participation	P18-P19
Dissemination policy: trial results	#31a	Plans for investigators and sponsor to communicate trial results to participants, healthcare professionals, the public, and other relevant groups (eg, via publication, reporting in results databases, or other data sharing arrangements), including any	P19

For peer review only

		publication restrictions	
Dissemination policy: authorship	#31b	Authorship eligibility guidelines and any intended use of professional writers	P19
Dissemination policy: reproducible research	#31c	Plans, if any, for granting public access to the full protocol, participant-level dataset, and statistical code	P19

Appendices

Informed consent materials	#32	Model consent form and other related documentation given to participants and authorised surrogates	Supplementary 1 and 2
Biological specimens	#33	Plans for collection, laboratory evaluation, and storage of biological specimens for genetic or molecular analysis in the current trial and for future use in ancillary studies, if applicable	NA

None The SPIRIT Explanation and Elaboration paper is distributed under the terms of the Creative Commons Attribution License CC-BY-NC. This checklist can be completed online using https://www.goodreports.org/, a tool made by the EQUATOR Network in collaboration with Penelope.ai

For peer review only

BMJ Open

Vitrectomy combined with lens capsule flap transplantation in the treatment of high myopia macular hole retinal detachment: study protocol for a prospective randomized controlled trial

Journal:	*BMJ Open*
Manuscript ID	bmjopen-2022-064299.R1
Article Type:	Protocol
Date Submitted by the Author:	29-Jun-2022
Complete List of Authors:	Gong, Qiaoyun; Shanghai General Hospital, Department of Ophthalmology<br>Ye, Luyao; Shanghai General Hospital, Department of Ophthalmology<br>Wu, Xia; Shanghai General Hospital, Department of Ophthalmology<br>Xue, Lin; Shanghai General Hospital, Department of Ophthalmology<br>Zhou, Hao; Shanghai General Hospital, Department of Ophthalmology<br>Fan, Ying; Shanghai General Hospital, Department of Ophthalmology<br>Xu, Xun; Shanghai General Hospital, Ophthalmology<br>Wang, Weijun; Shanghai General Hospital, Department of Ophthalmology<br>Qian, Tianwei; Shanghai General Hospital, Department of Ophthalmology
**Primary Subject Heading**:	Ophthalmology
Secondary Subject Heading:	Ophthalmology, Surgery
Keywords:	Vetreoretinal < OPHTHALMOLOGY, Ophthalmology < SURGERY, Medical retina < OPHTHALMOLOGY

SCHOLARONE™
Manuscripts

Vitrectomy combined with lens capsule flap transplantation in the treatment of high myopia macular hole retinal detachment: study protocol for a prospective randomized controlled trial

Qiaoyun Gong[1-5], Luyao Ye[1-5], Xia Wu[1-5], Lin Xue[1-5], Hao Zhou[1-5], Ying Fan[1-5], Xun Xu[1-5], Weijun Wang[1-5], Tianwei Qian[1-5]

[1] Department of Ophthalmology, Shanghai General Hospital, Shanghai Jiao Tong University, Shanghai, China

[2] National Clinical Research Center for Eye Diseases, Shanghai, China

[3] Shanghai Key Laboratory of Ocular Fundus Diseases, Shanghai, China

[4] Shanghai Engineering Center for Visual Science and Photomedicine, Shanghai, China

[5] Shanghai Engineering Center for Precise Diagnosis and Treatment of Eye Disease, Shanghai, China

Corresponding author: Tianwei Qian
100 Haining Road, Hongkou District, Shanghai 200080, China
Tel: +86 (0) 15201955168 Fax: 021-63240090
E-mail: qtw6180@126.com

Word count: 3919

For peer review only - http://bmjopen.bmj.com/site/about/guidelines.xhtml

Abstract

Introduction Vitrectomy combined with internal limiting membrane (ILM) peeling, flap or tamponade is widely used in the treatment of macular diseases, such as macular hole (MH) and high myopia macular hole retinal detachment (HMMHRD). However, movement of the ILM to a suitable position to prevent displacement is a difficult operation. Improving visual function after surgery remains controversial. Compared to ILM, the thicker and more flexible lens capsule is easy to obtain and operate. Previous studies have confirmed the effectiveness of lens capsule flap in the treatment of MH. This study aims to evaluate the efficacy and safety of vitrectomy combined with lens capsule flap transplantation in the treatment of HMMHRD.

Methods and analysis This single-centre, single-blind, prospective, randomised clinical trial will include 54 patients with HMMHRD who will first undergo phacoemulsification and intraocular lens implantation and then vitrectomy combined with lens capsule flap transplantation (experimental group) or ILM tamponade (control group). Study participants will be randomly allocated in a 1:1 ratio to experimental and control groups. Follow-up will be conducted 1, 3, and 7 days and 1, 3, and 6 months after surgery in both groups. Necessary examinations will be performed at each follow-up visit. Measurement outcomes include postoperative situation of macular hole closure, best corrected visual acuity, macular retinal function, and macular retinal sensitivity. The primary outcome is Type I closure rate of MH 6 months after operation. Intergroup comparisons of the proportions of patients with Type I closure of MH will be performed with Fisher's exact test.

Ethics and dissemination Full ethics approval for this study was obtained from the Ethics Committee of Shanghai General Hospital, Shanghai Jiaotong University, Shanghai, China. The outcomes of the trial will be disseminated through peer-reviewed journals and at scientific conferences.

Trial registration number ChiCTR2200057836

For peer review only - http://bmjopen.bmj.com/site/about/guidelines.xhtml

For peer review only

Strengths and limitations of this study

- This is the first randomised controlled trial investigating the efficacy and safety of vitrectomy combined with lens capsule flap transplantation in the treatment of high myopia macular hole retinal detachment (HMMHRD).
- In addition to anatomical measurement, functional measurements, including best corrected visual acuity, macular retinal function, and macular retinal sensitivity, are also used to evaluate the postoperative efficacy.
- Based on whether the macular hole reaches 400 μm, this trial will adopt the stratified randomization method through the central random system.
- Strict quality control measures, including adequate concealment of randomised group allocations, will be implemented.
- Difference in preoperative lens opacity affecting the baseline visual acuity of the two groups and difficult evaluation of vitreoretinal surgery are the limitations of this trial.

For peer review only

For peer review only - http://bmjopen.bmj.com/site/about/guidelines.xhtml

Introduction

High myopia is a common cause of vision loss. Globally, about 163 million individuals (2.7% of the world population) suffered from high myopia in 2000. With the increasing prevalence of HM, an estimated 1 billion individuals (9.8% of the world population) will suffer from high myopia in 2050.[1] Patients with high myopia have a higher risk of ocular disorders, such as cataract and glaucoma, than those without high myopia. In the field of the fundus, high myopia is associated with a series of pathological complications, including posterior staphyloma, myopic macular schisis (MMS), preretinal membrane, chorioretinal dystrophy, macular hole (MH), and retinal detachment (RD).[2-4] In patients with high myopia, about 6% of patients will develop MH, which is about 10 times higher than in individuals without high myopia.[5] Patients with high myopia have a long ocular axis and a high risk of posterior scleral staphyloma. Continuous traction of the vitreous leads to the formation of macular holes. The liquid in the vitreous reaches beneath the retinal neuroepithelial layer through the macular holes to form subretinal fluid (SRF), which will lead to high myopia macular hole retinal detachment (HMMHRD).[6] HMMHRD can be clearly diagnosed by optical coherence tomography (OCT). Once it occurs, vision deteriorates drastically, reducing quality of life. It is one of the serious complications threatening the vision of patients with high myopia.[7, 8] Possible mechanisms of HMMHRD include vitreoretinal traction caused by epiretinal membrane, residual vitreous cortex, inner limiting membrane (ILM), and retinal vascular system,[9] which makes the vertical and tangential traction generated by the epiretinal membrane and ILM,[10, 11] and further weakens the adhesion between retinal neuroepithelial layer and pigment epithelial layer (RPE). The long axis reduces the extension of the retina, resulting in the relative shortening of the retina, and subsequent retinal splitting and detachment.[12]

The key to the treatment of HMMHRD is to restore the normal anatomical structure of the macular region, improve the continuous traction state of the vitreous to the retina, and induce retinal reattachment. The main surgical procedures include extrascleral surgery (scleral buckling, macular banding, and posterior scleral reinforcement), intraocular surgery (vitrectomy), and combined intraocular and extraocular surgery. Currently, vitreoretinal

For peer review only - http://bmjopen.bmj.com/site/about/guidelines.xhtml

surgery (VRS) is the mainstream surgical method for the treatment of HMMHRD. Among them, pars plana vitrectomy (PPV) can relieve the mechanical traction of the vitreous cortex on the macula, restore the anatomical structure, effectively reduce retinal oedema and facilitate fluid exchange, and promote the recovery of retinal function in the macular region. It is one of the most routinely performed operations.[13] However, the abnormalities of the anatomical structure in high myopia, such as long ocular axis, retinal choroidal atrophy at the posterior pole, posterior scleral staphyloma, and "macular white hole", lead to increased surgical difficulty, resulting in low retinal reduction and hole closure rate and high recurrence rate in patients with HMMHRD after simple vitrectomy. In addition, scar healing, retinal interlayer structure disorder, RPE atrophy, and ganglion cell damage occur in macular holes after operation, which lead to unsatisfactory visual function recovery, even if retinal anatomical reduction was achieved.[14, 15] Therefore, to improve the success rate of surgery and preserve the visual function of patients as much as possible, combined macular interface surgery is needed after vitrectomy removes the traction of the vitreous to the macula. The combination of surgical methods, such as ILM peeling, ILM flap, and ILM tamponade, are currently in practice. Improving the visual function of patients based on improving the reduction rate is the key to the selection of macular interface surgery for HMMHRD.[16, 17]

Although these operations can improve the healing rate of macular hole, obtaining the inner limiting membrane tissue at the edge in patients with high myopia remains challenging. The challenge is due to the problems of posterior scleral staphyloma, retinal choroidal atrophy, and difficulty in identifying the inner limiting membrane.[18, 19] Moreover, because the ILM is originally soft, under the influence of the long axis of high myopia, ILM softens further under traction. Therefore, moving the ILM to a suitable position to prevent displacement is difficult.[19, 20] As a result, the role of improving visual function post-surgery remains controversial. Therefore, the choice of operation mode of macular interface is particularly important.

In 2019, Grewal *et al*[21] first reported that the lens capsule was transplanted to the macular hole for the treatment of refractory macular hole. Peng *et al*[22, 23] used an autologous

For peer review only - http://bmjopen.bmj.com/site/about/guidelines.xhtml

or allogeneic lens capsule to treat refractory macular hole. The healing rate can reach 90% and 96%, and the visual acuity of patients can be improved. Similar to the ILM, the lens capsule is a kind of basement membrane. Although the lens capsule is thicker than the ILM, it is quite flexible. Therefore, positioning the lens capsule at a designated place in the retina is easier than the ILM. For most patients who need surgery for HMMHRD, the degree of lens turbidity reaches the surgical indication of cataract phacoemulsification. Therefore, most doctors will choose to complete the operation together with cataract surgery to easily obtain the capsule tissue. The mechanism of lens capsule transplantation in the treatment of macular hole is similar to that of ILM. The lens capsule can be used as a scaffold to promote the proliferation and migration of Müller cells in the retina and promote the healing of macular holes. The study found that the lens capsule as a stent still existed and did not become thinner after nearly 3 years of follow-up.[23] Appropriate size and reasonable placement of lens capsule graft is the key to successful surgery.[23] Mastery of certain surgical skills is necessary to fill the lens capsule at the macular hole. Moreover, lens capsule transplantation cannot only promote the healing of macular hole but also improve the vision of patients.[20, 24, 25] In addition, lens epithelial cells (LECs) on the anterior capsule flap can be removed using distilled water, resulting in low antigenicity.[23] In the actual operation of capsule transplantation, Chen *et al*[16, 20] reported that the lens capsule is easier to process and insert MH than ILM, making it possible to vigorously promote this technology.

Based on the advantages of easy access, easy operation of lens capsule, combined with the clinical prognosis of high retinal reattachment rate and better improvement of visual function found in the capsule transplantation group in the previous study, our team plans to conduct a prospective randomized controlled trial of vitrectomy combined with lens capsule flap transplantation in the treatment of HMMHRD to explore the efficacy and safety of this operation in retinal reattachment and functional recovery after the treatment of HMMHRD.

Methods and analysis

Overview

For peer review only - http://bmjopen.bmj.com/site/about/guidelines.xhtml

This is a single-blind, prospective, and randomised clinical trial (RCT) being conducted at Shanghai General Hospital affiliated with Shanghai Jiaotong University. The patients have a clear diagnosis of HMMHRD and require vitrectomy. Our hypothesis is that vitrectomy combined with lens capsule flap transplantation in the treatment of HMMHRD can achieve a higher proportion of retinal reattachment and better functional recovery. After comprehensive ophthalmic and physical examinations, the patients will be randomised into an experimental group and control group to receive treatment.

For peer review only

Objective

The study aims to evaluate the efficacy and safety of vitrectomy combined with lens capsule flap transplantation in retinal reattachment and functional recovery in patients with HMMHRD.

Trial design

The prospective, single-centre RCT will enrol 54 participants that will be randomised in a 1:1 ratio to the experimental group (vitrectomy combined with lens capsule flap transplantation) or control group (vitrectomy combined with ILM tamponade). The patient's ophthalmic and systemic medical history and first examination results at admission will be recorded during the screening visit for the trial. Vitrectomy combined with lens capsule flap transplantation or ILM tamponade will be performed by one experienced surgeon. The protocol (date: 24/06/2022, version: 1.1) was developed and updated in accordance with the Consolidated Standards of Reporting Trials guidelines. This protocol was written in accordance with the Standard Protocol Items: Recommendations for Interventional Trials guidelines[26] and was approved by the Institutional Review Board of Shanghai General Hospital (Supplementary 1, approved ID: 2022SQ284.1). The trial has been registered with the Chinese Clinical Trial Registry (ChiCTR, http://www.chictr.org.cn/, No. ChiCTR2200057836). Any protocol modifications will be communicated to the participants and trial registry. Trial results will be disseminated on trial registration (ChiCTR) or through a publication. Participants have the right to withdraw their consent and discontinue participation at any time. A flowchart of the trial design is shown

For peer review only - http://bmjopen.bmj.com/site/about/guidelines.xhtml

in Figure 1.

Recruitment

Participants will be recruited by the surgeon during hospitalisation without any other form of advertisement. Interested patients will be invited to discuss the benefits and potential risks related to the vitrectomy combined with lens capsule flap transplantation. Participants meeting all the inclusion criteria will be fully informed of their responsibilities and all procedures involved in the trial. They will be asked to sign the written informed consent before enrolment. The English translation of informed consent can be seen in Supplementary 2.

Inclusion criteria

The inclusion criteria are as follows: (1) Patients ≥50 years old who were diagnosed as HMMHRD by indirect ophthalmoscopy, macular OCT, fundus photography, and B-ultrasound examination; (2) equivalent spherical dioptre ≤ –6.00 D or axis length ≥ 26 mm; (3) MH as main cause of RD; (4) turbid lens without obvious calcification in the lens capsule, requiring combination with cataract surgery; (5) the range of RD within the retinal vascular arch of the posterior pole; and (6) consent to undergo surgical treatment and regular follow-up and sign relevant informed consent.

Exclusion criteria

Exclusion criteria are as follows: (1) Patients with RD with other types of holes except MH, choroidal detachment, obvious proliferative vitreoretinopathy, intraocular haemorrhage, severe cataract, glaucoma, retinal vascular inflammation or obstruction, macular degeneration, macular neovascularization, macular haemorrhage, pathological myopia, and macular atrophy; (2) patients with previous history of intraocular surgery (including PPV and intravitreal drug injection); (3) patients whose general condition cannot tolerate the operation; (4) patients who cannot cooperate with the examination and follow-up for <6 months; (5) patients unwilling to undergo concurrent vitrectomy and cataract surgery; (6) patients with concomitant diseases or

For peer review only - http://bmjopen.bmj.com/site/about/guidelines.xhtml

conditions of the target eye or whole body (including malignant hypertension; AIDS; malignant tumour; serious mental, cardiovascular, neurologic, respiratory, digestive, or other systemic disease; long-term use of hormones; and immunodeficiency after heart stenting or organ transplantation); (7) patients with diabetes with poor blood glucose control; (8) patients with poor communication skills and compliance; (9) patients without the ability to follow the postoperative position; and (10) participation in other trials.

Withdrawal criteria

Withdrawal criteria are as follows: (1) loss to follow-up; (2) withdrawal of informed consent; (3) participation in other clinical studies during the study; (4) emergence of other eye diseases during the study; (5) need of other eye treatments during the study; (6) Decision by researchers to withdraw participant due to safety concerns (7) Decision by researchers to withdraw patient due to unsuitability of treatment methods (including remedial treatment) for patient.

Randomisation and blinding

The stratified randomisation method will be adopted in allocating participants to the two groups in a 1:1 ratio using the central random system. The stratification factors are as follows: 1) macular hole size > 400 μm and 2) macular hole size ≤ 400 μm. Except the surgeon, all researchers, including the outcome assessors, statisticians, and data analysts, will be blinded to group assignment, but providers of the intervention/control will be informed by necessity. Vitrectomy combined with lens capsule flap transplantation or ILM tamponade will be performed by one experienced surgeon. Before the trial, researchers will be fully trained in the randomisation procedure and made aware of their individual responsibilities. The successful implementation and maintenance of the randomisation and blinding method will be validated.

Interventions

All participants who meet the inclusion criteria of this study and do not meet any of the exclusion criteria can be included in this study. Before the surgery, the patients should receive

For peer review only - http://bmjopen.bmj.com/site/about/guidelines.xhtml

complete preoperative ophthalmic examination, including best corrected visual acuity (BCVA)，slit lamp inspection ，axial length ，presence of staphyloma ，myopia dioptres ，intraocular pressure (IOP)，ocular B-ultrasound，OCT，multifocal electrophysiology (mfERG)，MP-1 microperimeter，fluorescence fundus angiography (FFA) and indocyanine green angiography (ICGA). After passing the screening, the participants who provided written informed consent will be randomly assigned to two parallel trial groups in a 1:1 ratio according to the central randomization system. The patients will undergo vitrectomy combined with lens capsule flap transplantation or ILM tamponade and regular post-surgery follow-up. Participants will be blinded to the specific surgical methods. The timeline of data collection is shown in Table 1.

For peer review only - http://bmjopen.bmj.com/site/about/guidelines.xhtml

Table 1. Outcome measurements and data collection at each follow-up timepoint

Phase		Screen	Preoperative examination	Postoperative follow-up					
Follow-up		1	2	3	4	5	6	7	14
Time points		Within 1 week before operation	One day before operation	1 day after operation	3 days after operation	7 days after operation	1 month after operation	3 months after operation	6 months after operation
Informed consent		●							
Inclusion and exclusion criteria		●							
Withdrawal criteria			●	●	●	●	●	●	●
Medical history collection		●							
Ophthalmic examination	BCVA	●	●	●	●	●	●	●	●
	Slit lamp inspection	●	●	●	●	●	●	●	●
	Axial length	●						●	●
	Presence of staphyloma	●						●	●
	Myopia dioptres	●						●	●
	IOP	●	●	●	●	●	●	●	●
	Ocular B-ultrasound	●	●	●	●	●	●	●	●
	OCT	●	●	●	●	●	●	●	●

For peer review only - http://bmjopen.bmj.com/site/about/guidelines.xhtml

	mfERG	●						●	●
	MP-1 microperimeter	●						●	●
	FFA & ICGA	●							●
Systemic examination	Vital signs	●	●	●	●	●	●	●	●
	Physical examination	●							●
Laboratory examination	routine blood test	●	●				●		●
	Blood biochemistry	●					●		●
	Coagulation function	●							●
	Urinalysis	●							●
	Immune and infection measurements	●				●	●		●
	Electrocardiogram	●							●
	Chest CT	●							●
Adverse events recorded		●	●	●	●	●	●	●	●

BCVA, best corrected visual acuity; IOP, intraocular pressure; OCT, optical coherence tomography; mfERG, multifocal electrophysiology; FFA, fluorescence fundus angiography; ICGA, indocyanine green angiography.

For peer review only

Experimental group

Patients will first undergo phacoemulsification and intraocular lens implantation. During this operation, the lens capsule flap will be obtained and placed in sterile distilled water for at least 30 minutes. Thereafter, the 23-gauge vitrectomy through the pars plana will be performed, and the ILM will be stained with brilliant blue. After brilliant blue staining, the ILM in the macular area will be completely removed with intraocular forceps. Then the small piece of lens capsule flap is moved into the vitreous cavity with microforceps trough the 23-gauge trocar. The trimming of lens capsule flap will be performed in the vitreous cavity, and the lens capsule flap can be directly placed above the MH surface. The capsular flap should be trimmed by vitrectomy cutter as round as possible, and its diameter is about twice that of the macular hole. Afterwards, the lens capsule flap will need to be inserted into the macular hole to complete gas-liquid exchange. In this process, the operator will ensure that the capsule flap is always right under the macular hole. Finally, silicone oil (Oxane 5700 Bausch & Lomb, Kingston-upon-Thames, UK) will be injected into the vitreous cavity.

Control group

Patients will first undergo phacoemulsification and intraocular lens implantation. Thereafter, 23-gauge vitrectomy through the pars plana will be performed. After complete vitreous removal, ILM will be stained with brilliant blue. After brilliant blue staining, the ILM of the retina in the brilliant blue staining area will be directly clamped, and the ILM ring in the area will be removed about 1.5-disc diameters away from the macular hole. Care will be taken to prevent peeling off the ILM valve at the edge of the macular hole. Thereafter, the free ILM flap will be turned over and filled into the MH. During the operation, accurate filling of ILM into the MH will be confirmed severally. After complete gas-liquid exchange, silicone oil (Oxane 5700 Bausch & Lomb) will be injected into the vitreous cavity.

Postoperative management

All patients will be administered with anti-inflammatory and anti-infective treatment after

For peer review only - http://bmjopen.bmj.com/site/about/guidelines.xhtml

operation. During hospitalisation, IOP will be monitored 2–3 times daily. If IOP increases, it will be reduced. In case it is not controlled, mannitol will be administered intravenously to control IOP. If necessary, anterior chamber puncture, aspiration, and drainage or glaucoma surgery will be performed.

Outcome measurements

Follow-up will be conducted at 1, 3, and 7 days and 1, 3, and 6 months after surgery in both groups. Necessary examinations will be performed at each follow-up visit.

Anatomical measurement

OCT will be used to assess the retina and macula before and after surgery. According to the postoperative situation of retinal reattachment and macular hole closure, the surgical result will be classified as: (1) Type I closure: the two ends of the macular hole are closely connected and close to the RPE layer without any obvious exposure of the RPE layer and subretinal fluid (SRF); (2) Type II closure: the two ends of the macular hole are not connected, and the edge of the hole is closely attached to the RPE layer with a distance between the two ends. The exposed RPE layer can be observed without any obvious SRF; (3) Unclosed: the two ends of the macular hole are not connected, and the edge of the hole is still raised and tilted away from the RPE layer. The SRF can be observed below.[27, 28]

Functional measurement

Postoperative visual acuity, macular retinal function, and macular retinal sensitivity are all important functional outcomes. (1) Central vision: BCVA; (2) macular retinal function: mfERG collects the retinal amplitude information caused by specific stimulation, forms a three-dimensional topographic map, and collects P1 wave response density data for intuitive evaluation of macular retinal function; (3) Macular retinal sensitivity: MP-1 microperimetry is used to examine the micro visual field of the macula.

For peer review only

For peer review only - http://bmjopen.bmj.com/site/about/guidelines.xhtml

Primary outcome

The primary outcome is Type I closure rate of MH 6 months after operation.

Secondary outcomes

The following indicators will be used to comprehensively assess anatomical outcome: (1) Type I closure rate of macular hole 3 months after operation; (2) type II closure rate of macular hole 6 months after operation; and (3) type II closure rate of macular hole 3 months after operation. Functional outcomes will be assessed as follows: (1) Changes in BCVA 3 and 6 months after operation compared to baseline; (2) changes in P1 wave response density in the macular region 3 and 6 months after operation compared to baseline; and (3) changes in macular retinal sensitivity 3 and 6 months after operation compared to baseline.

Furthermore, the number of adverse events and reactions, abnormality of ophthalmic examination, abnormal systemic symptoms, and laboratory results will be recorded at each follow-up visit.

Data management and safety monitoring board

Basic demographic information and data on history of ocular diseases will be collected pre-intervention. All the data in this study will be collected from the clinical information system of our hospital and recorded in the case report format (CRF) in electric data collection (EDC) system developed by Shanghai General Hospital. Every participant included will be distinguished with a study identifier and initial without full name. An electronic password-protected document containing all the information from the CRFs will be set up for statistical analysis. Relevant documents will be stored at the Department of Ophthalmology of Shanghai General Hospital after the study. The confidentiality and safety of participants' data are guaranteed. All the data will be reserved for 5 years for the purpose of the designated scientific study.

To ensure the integrity of the RCT and protect the rights and health of the participants, a Data and Safety Monitoring Board will be constituted. As an independent advisory group, it will

For peer review only - http://bmjopen.bmj.com/site/about/guidelines.xhtml

ensure the scientific integrity and ethical standards of the RCT and be responsible for data evaluation during the study period. The Data and Safety Monitoring Board will be developed in accordance with the Operational Guidelines for the Establishment and Functioning of Data and Safety Monitoring Boards of the WHO. Researchers will be trained to collect good quality data, promote participant retention, and complete follow-up. Data analysts will be trained on data entry, coding, security, and storage. Statisticians will provide training on data assessment and statistical analysis.

Safety and combined operation-related adverse events

Possible phacoemulsification and intraocular lens implantation combined with vitrectomy complications include intraocular haemorrhage, suprachoroidal haemorrhage, endophthalmitis, uveitis, intraocular lens dislocation, and corneal endothelial decompensation. Lens capsule flap transplantation may increase the risk of rejection, although the capsule flap will be immersed in sterile distilled water for 30 minutes. However, to date, no adverse reactions caused by lens capsule flap transplantation for the treatment of MH have been reported. During the trial, we will record and analyse any surgery-related adverse events (including the type and number) in each group and possible causes. Patients will receive appropriate intervention for any adverse events that occur during the treatment and follow-up phases. Severe adverse events will be reported immediately to the primary investigator and affected participants will be withdrawn from the study.

Patients and public involvement

Neither the patients nor the public will be involved in the design of the study; nor will they be involved in its conduct (including outcome measurements). No attempt will be made to assess the burden of the intervention on the patients by themselves. Results excluding personal data will be made available on request.

Sample size

For peer review only - http://bmjopen.bmj.com/site/about/guidelines.xhtml

Although no RCT of lens capsule flap transplantation in the treatment of HMMHRD has been reported, according to the existing published papers[23, 29] related to this study, the Type I closure rate in the experimental group was expected to be 96.0%, whereas the rate in the control group was should be 65.0%. An a priori power analysis conducted using a two-tailed test (power = 0.8) at the 5% significance level indicated that 21 participants are required in each group. Considering the attrition rate of 20%, the sample size in each group was determined as 27. With the 1:1 allocation, 54 participants will be recruited.

Statistical analysis

SPSS (version 22.0; SPSS, Inc., Chicago, IL, USA) and GraphPad Prism (version 8, GraphPad software Inc., San Diego, CA, USA) will be used for data processing and analyses. Categorical variables will be expressed as numbers and frequencies, and between-group differences will be evaluated with Fisher's exact test. Continuous variables will be expressed as the mean ± standard deviation (SD). The Kolmogorov-Smirnov test will be used to determine whether continuous variables are normally distributed. The student's t test will be used for normally distributed data, and the Wilcoxon signed-rank test will be used for non-normally distributed data. All statistical tests will be two-sided with $P < 0.05$ considered statistically significant.

Ethics and dissemination

This RCT has been designed in accordance with the principles of the Declaration of Helsinki. The trial protocol has been approved by the Shanghai General Hospital Institutional Review Board (Approval ID: 2022SQ284.1). It is registered on the primary registry of the WHO registry network (Chinese Clinical Trial Registry). Signed consent will be obtained from each patient after they have been informed of the study procedures, possible risks, and their right to withdraw from the trial. The results of the trial without personal data will be directly communicated to participants and to the public through peer-reviewed publications and conference presentations. Standard and on-site screening are being conducted by an experienced retinal surgeon. Anaesthesiologists will monitor the whole surgical process.

For peer review only - http://bmjopen.bmj.com/site/about/guidelines.xhtml

Trial status

This study is currently in the preparatory stage. The first participant will be enrolled in April 2023. The projected completion date is March 2025.

Acknowledgements

The trial will be conducted at Shanghai General Hospital. The authors thank the patients and their family members, as well as the nurses, pharmacy staff, fellows, statisticians, and clinicians for making this study possible.

Contributors

TQ, QG, LY, YF, WW, and XX participated in trial design. TQ, QG, LX, YF, and WW designed the data analysis plan. LY, XW, HZ, and WW collected the information needed to conduct this trial. QG, WW, and TQ wrote the first draft of the manuscript. All authors reviewed and revised the manuscript and gave final approval for the publication of this study protocol.

Funding

This study was supported by Special Project for Clinical Research in Health Industry of Shanghai Health Commission (No. 20214Y0045).

Competing interests

None declared.

Ethics approval

The study protocol has been approved by the Shanghai General Hospital Institutional Review Board (Approved ID: 2022SQ284.1).

For peer review only

For peer review only - http://bmjopen.bmj.com/site/about/guidelines.xhtml

Provenance and peer review

Not commissioned; externally peer reviewed.

Data Availability Statement

After the research is completed, we will publish results in peer-reviewed journals. Data from this study may be requested 5 years after the completion of the primary endpoint by contacting the corresponding author upon reasonable request.

For peer review only

For peer review only - http://bmjopen.bmj.com/site/about/guidelines.xhtml

References

1. Holden BA, Fricke TR, Wilson DA, Jong M, Naidoo KS, Sankaridurg P, Wong TY, Naduvilath TJ, Resnikoff S: **Global Prevalence of Myopia and High Myopia and Temporal Trends from 2000 through 2050**. *Ophthalmology* 2016, **123**(5):1036-1042.
2. Henaine-Berra A, Zand-Hadas IM, Fromow-Guerra J, García-Aguirre G: **Prevalence of macular anatomic abnormalities in high myopia**. *Ophthalmic surgery, lasers & imaging retina* 2013, **44**(2):140-144.
3. Rino F, Elena Z, Ivan M, Paolo B, Barbara P, Federica R: **Lamellar macular hole in high myopic eyes with posterior staphyloma: morphological and functional characteristics**. *Graefe's archive for clinical and experimental ophthalmology = Albrecht von Graefes Archiv fur klinische und experimentelle Ophthalmologie* 2016, **254**(11):2141-2150.
4. Chang L, Pan CW, Ohno-Matsui K, Lin X, Cheung GC, Gazzard G, Koh V, Hamzah H, Tai ES, Lim SC *et al*: **Myopia-related fundus changes in Singapore adults with high myopia**. *American journal of ophthalmology* 2013, **155**(6):991-999.e991.
5. Wilczyński T, Heinke A, Niedzielska-Krycia A, Jorg D, Michalska-Małecka K: **Optical coherence tomography angiography features in patients with idiopathic full-thickness macular hole, before and after surgical treatment**. *Clinical interventions in aging* 2019, **14**:505-514.
6. Liu B, Chen S, Li Y, Lian P, Zhao X, Yu X, Li T, Jin C, Liang X, Huang SS *et al*: **Comparison of macular buckling and vitrectomy for the treatment of macular schisis and associated macular detachment in high myopia: a randomized clinical trial**. *Acta ophthalmologica* 2020, **98**(3):e266-e272.
7. Lichtwitz O, Boissonnot M, Mercié M, Ingrand P, Leveziel N: **Prevalence of macular complications associated with high myopia by multimodal imaging**. *Journal francais d'ophtalmologie* 2016, **39**(4):355-363.
8. Haarman AEG, Tedja MS, Brussee C, Enthoven CA, van Rijn GA, Vingerling JR, Keunen JEE, Boon CJF, Geerards AJM, Luyten GPM *et al*: **Prevalence of Myopic Macular Features in Dutch Individuals of European Ancestry With High Myopia**. *JAMA ophthalmology* 2022, **140**(2):115-123.
9. Kamal-Salah R, Morillo-Sanchez MJ, Rius-Diaz F, Garcia-Campos JM: **Relationship between paravascular abnormalities and foveoschisis in highly myopic patients**. *Eye (London, England)* 2015, **29**(2):280-285.
10. Gao X, Guo J, Meng X, Wang J, Peng X, Ikuno Y: **A meta-analysis of vitrectomy with or without internal limiting membrane peeling for macular hole retinal detachment in the highly myopic eyes**. *BMC ophthalmology* 2016, **16**:87.
11. Gandorfer A, Scheler R, Haritoglou C, Schumann R, Nentwich M, Kampik A: **Pathology of the macular hole rim in flat-mounted internal limiting membrane specimens**. *Retina (Philadelphia, Pa)* 2009, **29**(8):1097-1105.
12. Gao Y, Ruan T, Chen N, Yu B, Xing X, Du Q, Qi Y, Li J: **A Comparison of Face-Down Positioning and Adjustable Positioning After Pars Plana Vitrectomy for**

For peer review only - http://bmjopen.bmj.com/site/about/guidelines.xhtml

Macular Hole Retinal Detachment in High Myopia. *Frontiers in medicine* 2022, **9**:780475.

13. Peng KL, Kung YH, Hsu CM, Chang SP, Tseng PL, Wu TT: **Surgical outcomes of centripetal non-fovea-sparing internal limiting membrane peeling for myopic foveoschisis with and without foveal detachment: a follow-up of at least 3 years**. *The British journal of ophthalmology* 2020, **104**(9):1266-1270.
14. Yamashiro K, Kinoshita-Nakano E, Ota T, Jingami Y, Nakata I, Hayashi H: **Floating flap of internal limiting membrane in myopic macular hole surgery**. *Graefe's archive for clinical and experimental ophthalmology = Albrecht von Graefes Archiv fur klinische und experimentelle Ophthalmologie* 2018, **256**(4):693-698.
15. Ohno-Matsui K, Jonas JB: **Posterior staphyloma in pathologic myopia**. *Progress in retinal and eye research* 2019, **70**:99-109.
16. Chen SN, Hsieh YT, Yang CM: **Multiple Free Internal Limiting Membrane Flap Insertion in the Treatment of Macular Hole-Associated Retinal Detachment in High Myopia**. *Ophthalmologica Journal international d'ophtalmologie International journal of ophthalmology Zeitschrift fur Augenheilkunde* 2018, **240**(3):143-149.
17. Michalewska Z, Michalewski J, Dulczewska-Cichecka K, Nawrocki J: **Inverted internal limiting membrane flap technique for surgical repair of myopic macular holes**. *Retina (Philadelphia, Pa)* 2014, **34**(4):664-669.
18. Morizane Y, Shiraga F, Kimura S, Hosokawa M, Shiode Y, Kawata T, Hosogi M, Shirakata Y, Okanouchi T: **Autologous transplantation of the internal limiting membrane for refractory macular holes**. *American journal of ophthalmology* 2014, **157**(4):861-869.e861.
19. Lai CC, Chen YP, Wang NK, Chuang LH, Liu L, Chen KJ, Hwang YS, Wu WC, Chen TL: **Vitrectomy with Internal Limiting Membrane Repositioning and Autologous Blood for Macular Hole Retinal Detachment in Highly Myopic Eyes**. *Ophthalmology* 2015, **122**(9):1889-1898.
20. Chen SN, Yang CM: **LENS CAPSULAR FLAP TRANSPLANTATION IN THE MANAGEMENT OF REFRACTORY MACULAR HOLE FROM MULTIPLE ETIOLOGIES**. *Retina (Philadelphia, Pa)* 2016, **36**(1):163-170.
21. Grewal DS, Charles S, Parolini B, Kadonosono K, Mahmoud TH: **Autologous Retinal Transplant for Refractory Macular Holes: Multicenter International Collaborative Study Group**. *Ophthalmology* 2019, **126**(10):1399-1408.
22. Peng J, Chen C, Jin H, Zhang H, Zhao P: **AUTOLOGOUS LENS CAPSULAR FLAP TRANSPLANTATION COMBINED WITH AUTOLOGOUS BLOOD APPLICATION IN THE MANAGEMENT OF REFRACTORY MACULAR HOLE**. *Retina (Philadelphia, Pa)* 2018, **38**(11):2177-2183.
23. Peng J, Chen C, Zhang H, Zhang L, Liu J, Ren J, Zhao P: **LONG-TERM SURGICAL OUTCOMES OF LENS CAPSULAR FLAP TRANSPLANTATION IN THE MANAGEMENT OF REFRACTORY MACULAR HOLE**. *Retina (Philadelphia, Pa)* 2021, **41**(4):726-734.

For peer review only - http://bmjopen.bmj.com/site/about/guidelines.xhtml

24. Gosala S, Manchikanty SM: **Inverted Internal Limiting Membrane Insertion for Macular Hole-Associated Retinal Detachment in High Myopia**. *American journal of ophthalmology* 2016, **166**:210-211.
25. Peng J, Chen C, Zhang L, Huang Y, Zhang H, Zheng Y, Ren J, Zou Y, Zhang X, Zhao P: **LENS CAPSULAR FLAP TRANSPLANTATION AS PRIMARY TREATMENT FOR CLOSURE OF LARGE MACULAR HOLES**. *Retina (Philadelphia, Pa)* 2022, **42**(2):306-312.
26. Chan AW, Tetzlaff JM, Gøtzsche PC, Altman DG, Mann H, Berlin JA, Dickersin K, Hróbjartsson A, Schulz KF, Parulekar WR *et al*: **SPIRIT 2013 explanation and elaboration: guidance for protocols of clinical trials**. *BMJ (Clinical research ed)* 2013, **346**:e7586.
27. Kang SW, Ahn K, Ham DI: **Types of macular hole closure and their clinical implications**. *The British journal of ophthalmology* 2003, **87**(8):1015-1019.
28. Imai M, Iijima H, Gotoh T, Tsukahara S: **Optical coherence tomography of successfully repaired idiopathic macular holes**. *American journal of ophthalmology* 1999, **128**(5):621-627.
29. Zheng Y, Kang M, Wang H, Liu H, Sun T, Sun X, Wang F: **Inverted internal limiting membrane insertion combined with air tamponade in the treatment of macular hole retinal detachment in high myopia: study protocol for a randomized controlled clinical trial**. *Trials* 2018, **19**(1):469.

For peer review only

For peer review only - http://bmjopen.bmj.com/site/about/guidelines.xhtml

Figure Legend

Figure 1 Flowchart of the trial design.

For peer review only

For peer review only - http://bmjopen.bmj.com/site/about/guidelines.xhtml

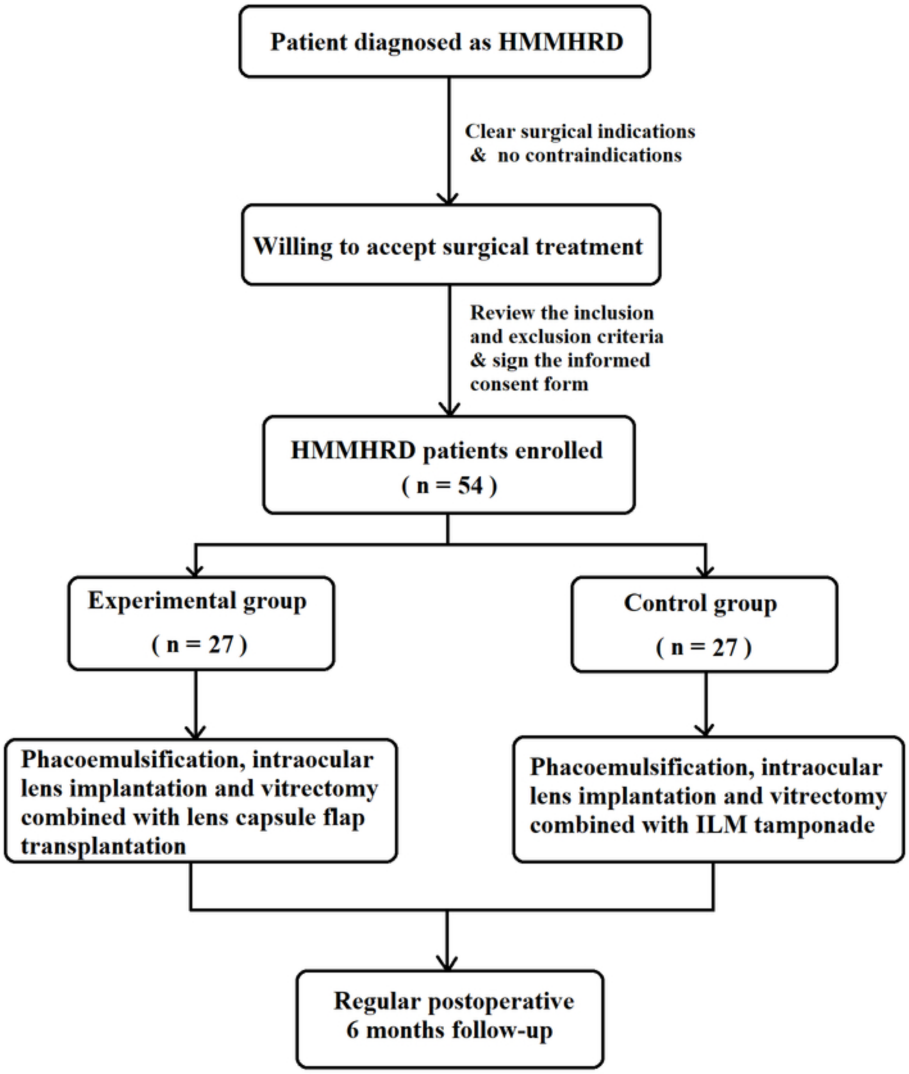

Flowchart of the trial design.

66x73mm (300 x 300 DPI)

Institutional Review Board of Shanghai General Hospital

Approval Letter of Shanghai General Hospital Institutional Review Board

**Approval No:** 2022SQ284.1	**Review date:** June 24, 2022	**Project No:** 2022SQ284.1	
**Study Protocol**	A prospective randomized controlled trial of vitrectomy combined with lens capsule flap transplantation in the treatment of high myopia macular hole retinal detachment		
**Project source**	Initiated by researchers		
**Principal investigator**	Tianwei Qian		
**Research Center**	Shanghai General Hospital	**Department**	Ophthalmology

Review documents (including version number)

(1) Study protocol (version: 1.1)

(2) Resume of principal investigator (version: 1.0)

(3) Informed consent (version: 1.1)

1. Review method

Rapid review

2. Results

Agree

3. The study will be subject to ongoing review by the ethics committee?

■ **Yes** ☐**No**

The censorship frequency is once every 12 months from the date of approval. The ethics committee has the right to change the frequency of continuous review according to the actual situation.

Institutional Review Board

Date: June 24, 2022

Address: No.100 Haining Road, Shanghai **Official E-mail:** shiyilunli@sina.com

Informed Consent Notification Page

Trial: A prospective randomized controlled trial of vitrectomy combined with lens capsule flap transplantation in the treatment of high myopia macular hole retinal detachment

Sponsor：Shanghai General Hospital

We invite you to participate in a study: A prospective randomized controlled trial of vitrectomy combined with lens capsule flap transplantation in the treatment of high myopia macular hole retinal detachment. Before you decide to participate in this trial, please read this informed consent carefully. If you have any questions you don't understand, you can ask the researcher in charge of the trial or members of the trial working group to explain any terms or materials you don't know.

1. Research background and purpose

1.1 Research background

Vitrectomy combined with internal limiting membrane (ILM) peeling, flap or tamponade is widely used in the treatment of macular diseases, such as macular hole (MH) and high myopia macular hole retinal detachment (HMMHRD). However, movement of the ILM to a suitable position to prevent displacement is a difficult operation. Improving visual function after surgery remains controversial. Compared to ILM, the thicker and more flexible lens capsule is easy to obtain and operate. Previous studies have confirmed the effectiveness of lens capsule flap in the treatment of MH.

1.2 Research purpose

This study aims to evaluate the efficacy and safety of vitrectomy combined with lens capsule flap transplantation in the treatment of HMMHRD.

2. Methods

2.1 Who can take part in the trial?

The inclusion criteria are as follows:

For peer review only - http://bmjopen.bmj.com/site/about/guidelines.xhtml

1） Patients ≥ 50 years old who were diagnosed as HMMHRD by indirect ophthalmoscopy, macular OCT, fundus photography, and B-ultrasound examination;

2） equivalent spherical dioptre ≤ –6.00 D or axis length ≥ 26 mm;

3） MH as main cause of RD;

4） turbid lens without obvious calcification in the lens capsule, requiring combination with cataract surgery;

5） the range of RD within the retinal vascular arch of the posterior pole;

6） consent to undergo surgical treatment and regular follow-up and sign relevant informed consent.

2.2 Who can't take part in the experiment?

1） patients with RD with other types of holes except MH, choroidal detachment, obvious proliferative vitreoretinopathy, intraocular haemorrhage, severe cataract, glaucoma, retinal vascular inflammation or obstruction, macular degeneration, macular neovascularization, macular haemorrhage, pathological myopia, and macular atrophy;

2） patients with previous history of intraocular surgery (including PPV and intravitreal drug injection)

3） patients whose general condition cannot tolerate the operation;

4） patients who cannot cooperate with the examination and follow-up for <6 months;

5） patients unwilling to undergo concurrent vitrectomy and cataract surgery;

6） patients with concomitant diseases or conditions of the target eye or whole body (including malignant hypertension; AIDS; malignant tumour; serious mental, cardiovascular, neurologic, respiratory, digestive, or other systemic disease; long-term use of hormones; and immunodeficiency after heart stenting or organ transplantation);

7） patients with diabetes with poor blood glucose control;

8） patients with poor communication skills and compliance;

9） patients without the ability to follow the postoperative position;

10） participation in other trials.

2.3 Trial design and sample size

For peer review only - http://bmjopen.bmj.com/site/about/guidelines.xhtml

For peer review only

The prospective, single-centre RCT will enrol 54 participants that will be randomised in a 1:1 ratio to the experimental group (vitrectomy combined with lens capsule flap transplantation) or control group (vitrectomy combined with ILM tamponade).

2.4 Interventions

2.4.1 Experimental group

Patients will first undergo phacoemulsification and intraocular lens implantation. During this operation, the lens capsule flap will be obtained and placed in sterile distilled water for at least 30 minutes. Thereafter, the 23-gauge vitrectomy through the pars plana will be performed, and the ILM will be stained with brilliant blue. After brilliant blue staining, the ILM in the macular area will be completely removed with intraocular forceps. Then the small piece of lens capsule flap is moved into the vitreous cavity with microforceps trough the 23-gauge trocar. The trimming of lens capsule flap will be performed in the vitreous cavity, and the lens capsule flap can be directly placed above the MH surface. The capsular flap should be trimmed by vitrectomy cutter as round as possible, and its diameter is about twice that of the macular hole. Afterwards, the lens capsule flap will need to be inserted into the macular hole to complete gas-liquid exchange. In this process, the operator will ensure that the capsule flap is always right under the macular hole. Finally, silicone oil (Oxane 5700 Bausch & Lomb, Kingston-upon-Thames, UK) will be injected into the vitreous cavity.

2.4.2 Control group

Patients will first undergo phacoemulsification and intraocular lens implantation. Thereafter, 23-gauge vitrectomy through the pars plana will be performed. After complete vitreous removal, ILM will be stained with brilliant blue. After brilliant blue staining, the ILM of the retina in the brilliant blue staining area will be directly clamped, and the ILM ring in the area will be removed about 1.5-disc diameters away from the macular hole. Care will be taken to prevent peeling off the ILM valve at the edge of the macular hole. Thereafter, the free ILM flap will be turned over and filled into the MH. During the operation, accurate filling of ILM into the MH will be confirmed severally. After complete gas-liquid exchange, silicone oil (Oxane 5700 Bausch & Lomb) will be injected into the vitreous cavity.

2.5 Research procedures

After fully informed and signed the informed consent, the subjects could enter the screening

For peer review only - http://bmjopen.bmj.com/site/about/guidelines.xhtml

period; In the screening period, the evaluation researchers evaluated whether they were included or not according to the inclusion/exclusion criteria. Only the qualified subjects can enter the study treatment period. The research doctor needs your cooperation in the implementation of relevant examination items to determine whether you meet the inclusion and exclusion criteria. These examinations are routine examinations for clinical diagnosis and treatment of this disease.

All participants who meet the inclusion criteria of this study and do not meet any of the exclusion criteria can be included in this study. After passing the screening, the participants who provided written informed consent will be randomly assigned to two parallel trial groups in a 1:1 ratio according to the central randomization system. The patients will undergo vitrectomy combined with lens capsule flap transplantation or ILM tamponade and regular post-surgery follow-up. Participants will be blinded to the specific surgical methods.

3. Research risks and benefits

3.1 Research risks

When your health condition is harmed by participating in this trial, the research doctor will take necessary medical measures. If you have any side effects or discomfort during the trial, it is essential that you report to the study doctor immediately. You may be given other drugs to control side effects. When participating in this study, you will need to provide some personal information, and we will take all necessary measures to ensure the confidentiality of the information.

3.2 Research benefits

Your participation may be helpful to further explore the efficacy and safety of vitrectomy combined with lens capsule flap transplantation in the treatment of HMMHRD.

4. Your rights

You have the right to decide whether to participate in the experiment. If you can't make a decision immediately, you have enough time to consider it. If necessary, you can discuss with relatives, friends and other people you trust before making a decision. If you decide not to participate in this trial, your relationship with the researcher and the sponsor will not be affected, you will not be discriminated against or retaliated, and your treatment and rights will not be affected. If you decide to participate in this trial, we hope you can complete the trial without special reasons, but you have the right to withdraw at any time during the trial. If you decide to quit, please let the researcher know in time.

During the experiment, you can know the information related to you at any time. If you have any

For peer review only - http://bmjopen.bmj.com/site/about/guidelines.xhtml

questions about this study, or if you feel unwell during the treatment, please contact the attending doctor. Contact doctor: Tianwei Qian 15201955168. And you have the right to consult about your rights or related risks.

5. Privacy and confidentiality

The personal information (such as name, gender, contact information, questionnaire, etc.) provided by you to the researcher may be known to the following persons or units in addition to the needs of normal research:

- Staff (inspectors, inspectors, etc.) of research funding institutions related to this experiment;
- State and local food and Drug Administration and other administrative agencies.

However, no one is allowed to disclose your personal information to others or other organizations without your permission. Except for researchers and administrative organizations, no other person or organization has the right to contact you about this experiment or provide you with information about this experiment directly.

The results of this experiment may be published in the form of academic papers, but your personal information will not appear in any publicly published documents.

6. Others

6.1 In case of the following situations, the researcher may withdraw you from the trial without your consent for your health::

- If you continue to participate in this trial, your risk may outweigh your benefit;
- You did not follow the guidance of the researcher and did not participate in the trial according to the research plan;
- Early termination of trial.

6.2 This informed consent is in duplicate, one for the researcher and one for you.

7. Compensation for injury caused by trial

If your injury is directly caused by participating in this trial, you do not have to pay the medical expenses for treatment, which will be borne by the researcher.

For peer review only - http://bmjopen.bmj.com/site/about/guidelines.xhtml

Informed Consent Page

Consent statement:

1. I have read the instructions to the subjects carefully and understood the relevant background of this trial. The researchers have explained the characteristics of the study and possible adverse reactions to me in detail, and answered my questions.

2. I know that if I refuse to participate in this trial, my treatment and rights will not be affected. After understanding all the contents of the instructions to the subjects and having fully considered them, I voluntarily participate in this trial.

3. I am willing to follow the instructions of the researchers and participate in the trial according to the research protocol. During the experiment, I have the right to withdraw at any time, but before I withdraw, I need to inform the researchers in time.

4. During the trial, if there are any discomfort symptoms, I will tell the researchers in time.

Signature of Subject:

/ /

Name (regular script) Signature Date

Signature of Researcher:

/ /

Name (regular script) Signature Date

Signature of agent/Guardian (if any):

The reason why the subject can't sign this page: ______

The relationship between the agent/guardian and the subject: ______

/ /

Name (regular script) Signature Date

For peer review only

For peer review only - http://bmjopen.bmj.com/site/about/guidelines.xhtml

Reporting checklist for protocol of a clinical trial.

Based on the SPIRIT guidelines.

Instructions to authors

Complete this checklist by entering the page numbers from your manuscript where readers will find each of the items listed below.

Your article may not currently address all the items on the checklist. Please modify your text to include the missing information. If you are certain that an item does not apply, please write "n/a" and provide a short explanation.

Upload your completed checklist as an extra file when you submit to a journal.

In your methods section, say that you used the SPIRITreporting guidelines, and cite them as:

Chan A-W, Tetzlaff JM, Gøtzsche PC, Altman DG, Mann H, Berlin J, Dickersin K, Hróbjartsson A, Schulz KF, Parulekar WR, Krleža-Jerić K, Laupacis A, Moher D. SPIRIT 2013 Explanation and Elaboration: Guidance for protocols of clinical trials. BMJ. 2013;346:e7586

		Reporting Item	Page Number
**Administrative information**			
Title	#1	Descriptive title identifying the study design, population, interventions, and, if applicable, trial acronym	P1
Trial registration	#2a	Trial identifier and registry name. If not yet	P2

For peer review only

		registered, name of intended registry	
Trial registration: data set	#2b	All items from the World Health Organization Trial Registration Data Set	P2
Protocol version	#3	Date and version identifier	P7
Funding	#4	Sources and types of financial, material, and other support	P18
Roles and responsibilities: contributorship	#5a	Names, affiliations, and roles of protocol contributors	P18
Roles and responsibilities: sponsor contact information	#5b	Name and contact information for the trial sponsor	P1
Roles and responsibilities: sponsor and funder	#5c	Role of study sponsor and funders, if any, in study design; collection, management, analysis, and interpretation of data; writing of the report; and the decision to submit the report for publication, including whether they will have ultimate authority over any of these activities	P18
Roles and responsibilities: committees	#5d	Composition, roles, and responsibilities of the coordinating centre, steering committee, endpoint adjudication committee, data management team, and other individuals or groups overseeing the trial, if	P15, P18

For peer review only

		applicable (see Item 21a for data monitoring committee)	
**Introduction**			
Background and rationale	#6a	Description of research question and justification for undertaking the trial, including summary of relevant studies (published and unpublished) examining benefits and harms for each intervention	P4-P6
Background and rationale: choice of comparators	#6b	Explanation for choice of comparators	P4-P6
Objectives	#7	Specific objectives or hypotheses	P7
Trial design	#8	Description of trial design including type of trial (eg, parallel group, crossover, factorial, single group), allocation ratio, and framework (eg, superiority, equivalence, non-inferiority, exploratory)	P7
**Methods: Participants, interventions, and outcomes**			
Study setting	#9	Description of study settings (eg, community clinic, academic hospital) and list of countries where data will be collected. Reference to where list of study sites can be obtained	P7

For peer review only

Eligibility criteria	#10	Inclusion and exclusion criteria for participants. If applicable, eligibility criteria for study centres and individuals who will perform the interventions (eg, surgeons, psychotherapists)	P8-P9
Interventions: description	#11a	Interventions for each group with sufficient detail to allow replication, including how and when they will be administered	P9-P10
Interventions: modifications	#11b	Criteria for discontinuing or modifying allocated interventions for a given trial participant (eg, drug dose change in response to harms, participant request, or improving / worsening disease)	P10-P12
Interventions: adherance	#11c	Strategies to improve adherence to intervention protocols, and any procedures for monitoring adherence (eg, drug tablet return; laboratory tests)	P10-P13
Interventions: concomitant care	#11d	Relevant concomitant care and interventions that are permitted or prohibited during the trial	P10-P13
Outcomes	#12	Primary, secondary, and other outcomes, including the specific measurement variable (eg, systolic blood pressure), analysis metric (eg, change from baseline, final value, time to event), method of aggregation (eg, median, proportion), and time point for each outcome. Explanation of the clinical relevance of chosen efficacy and harm outcomes is strongly recommended	P14-P15

For peer review only

Participant timeline	#13	Time schedule of enrolment, interventions (including any run-ins and washouts), assessments, and visits for participants. A schematic diagram is highly recommended (see Figure)	P7, P10-P12
Sample size	#14	Estimated number of participants needed to achieve study objectives and how it was determined, including clinical and statistical assumptions supporting any sample size calculations	P16
Recruitment	#15	Strategies for achieving adequate participant enrolment to reach target sample size	P8-P9
**Methods: Assignment of interventions (for controlled trials)**			
Allocation: sequence generation	#16a	Method of generating the allocation sequence (eg, computer-generated random numbers), and list of any factors for stratification. To reduce predictability of a random sequence, details of any planned restriction (eg, blocking) should be provided in a separate document that is unavailable to those who enrol participants or assign interventions	P9
Allocation concealment mechanism	#16b	Mechanism of implementing the allocation sequence (eg, central telephone; sequentially numbered, opaque, sealed envelopes), describing any steps to	P9-P10

For peer review only

		conceal the sequence until interventions are assigned	
Allocation: implementation	#16c	Who will generate the allocation sequence, who will enrol participants, and who will assign participants to interventions	P15-P16
Blinding (masking)	#17a	Who will be blinded after assignment to interventions (eg, trial participants, care providers, outcome assessors, data analysts), and how	P15-P16
Blinding (masking): emergency unblinding	#17b	If blinded, circumstances under which unblinding is permissible, and procedure for revealing a participant's allocated intervention during the trial	P15-P16
**Methods: Data collection, management, and analysis**			
Data collection plan	#18a	Plans for assessment and collection of outcome, baseline, and other trial data, including any related processes to promote data quality (eg, duplicate measurements, training of assessors) and a description of study instruments (eg, questionnaires, laboratory tests) along with their reliability and validity, if known. Reference to where data collection forms can be found, if not in the protocol	P15-P16
Data collection plan:	#18b	Plans to promote participant retention and complete	P15-P16

For peer review only

retention		follow-up, including list of any outcome data to be collected for participants who discontinue or deviate from intervention protocols	
Data management	#19	Plans for data entry, coding, security, and storage, including any related processes to promote data quality (eg, double data entry; range checks for data values). Reference to where details of data management procedures can be found, if not in the protocol	P15-P16
Statistics: outcomes	#20a	Statistical methods for analysing primary and secondary outcomes. Reference to where other details of the statistical analysis plan can be found, if not in the protocol	P14-P17
Statistics: additional analyses	#20b	Methods for any additional analyses (eg, subgroup and adjusted analyses)	P14-P17
Statistics: analysis population and missing data	#20c	Definition of analysis population relating to protocol non-adherence (eg, as randomised analysis), and any statistical methods to handle missing data (eg, multiple imputation)	P16-P17
**Methods: Monitoring**			
Data monitoring: formal committee	#21a	Composition of data monitoring committee (DMC); summary of its role and reporting structure; statement of whether it is independent from the sponsor and competing interests; and reference to	P15

For peer review only

		where further details about its charter can be found, if not in the protocol. Alternatively, an explanation of why a DMC is not needed	
Data monitoring: interim analysis	#21b	Description of any interim analyses and stopping guidelines, including who will have access to these interim results and make the final decision to terminate the trial	P15
Harms	#22	Plans for collecting, assessing, reporting, and managing solicited and spontaneously reported adverse events and other unintended effects of trial interventions or trial conduct	P15-P16
Auditing	#23	Frequency and procedures for auditing trial conduct, if any, and whether the process will be independent from investigators and the sponsor	P15-P16
**Ethics and dissemination**			
Research ethics approval	#24	Plans for seeking research ethics committee / institutional review board (REC / IRB) approval	P17
Protocol amendments	#25	Plans for communicating important protocol modifications (eg, changes to eligibility criteria, outcomes, analyses) to relevant parties (eg, investigators, REC / IRBs, trial participants, trial registries, journals, regulators)	P17

For peer review only

Consent or assent	#26a	Who will obtain informed consent or assent from potential trial participants or authorised surrogates, and how (see Item 32)	P17
Consent or assent: ancillary studies	#26b	Additional consent provisions for collection and use of participant data and biological specimens in ancillary studies, if applicable	P17
Confidentiality	#27	How personal information about potential and enrolled participants will be collected, shared, and maintained in order to protect confidentiality before, during, and after the trial	P17
Declaration of interests	#28	Financial and other competing interests for principal investigators for the overall trial and each study site	P18
Data access	#29	Statement of who will have access to the final trial dataset, and disclosure of contractual agreements that limit such access for investigators	P19
Ancillary and post trial care	#30	Provisions, if any, for ancillary and post-trial care, and for compensation to those who suffer harm from trial participation	P18
Dissemination policy: trial results	#31a	Plans for investigators and sponsor to communicate trial results to participants, healthcare professionals, the public, and other relevant groups (eg, via publication, reporting in results databases, or other data sharing arrangements), including any	P18

For peer review only

		publication restrictions	
Dissemination policy: authorship	#31b	Authorship eligibility guidelines and any intended use of professional writers	P18
Dissemination policy: reproducible research	#31c	Plans, if any, for granting public access to the full protocol, participant-level dataset, and statistical code	P18
**Appendices**			
Informed consent materials	#32	Model consent form and other related documentation given to participants and authorised surrogates	Supplementary 1 and 2
Biological specimens	#33	Plans for collection, laboratory evaluation, and storage of biological specimens for genetic or molecular analysis in the current trial and for future use in ancillary studies, if applicable	NA

None The SPIRIT Explanation and Elaboration paper is distributed under the terms of the Creative Commons Attribution License CC-BY-NC. This checklist can be completed online using https://www.goodreports.org/, a tool made by the EQUATOR Network in collaboration with Penelope.ai

For peer review only
